# Competition between a transmembrane helix on Scap and a membrane cholesterol regulates Scap–Insig interaction and SREBP activation

**DOI:** 10.1073/pnas.2525043123

**Published:** 2025-12-31

**Authors:** Blake C. Williams, Daniel L. Kober, Xiao-chen Bai, Daniel M. Rosenbaum, Arun Radhakrishnan

**Affiliations:** ^a^Department of Biophysics, University of Texas Southwestern Medical Center, Dallas, TX 75390; ^b^Department of Biochemistry, University of Texas Southwestern Medical Center, Dallas, TX 75390; ^c^Department of Molecular Genetics, University of Texas Southwestern Medical Center, Dallas, TX 75390

**Keywords:** cholesterol, Scap, SREBP, lipid metabolism

## Abstract

Cholesterol homeostasis in mammalian cells relies on the interaction between two endoplasmic reticulum (ER) sterol-sensing membrane proteins, Scap and Insig. Their interaction regulates activation of transcription factors called sterol regulatory element–binding proteins (SREBPs) that control genes for cholesterol biosynthesis and uptake. Previous studies suggested a model where cholesterol sensing by Scap involves communication across the ER membrane between two functional domains, a cholesterol-binding domain on the luminal side and a COPII-binding domain on the cytosolic side. When ER cholesterol is low, Scap binds COPII adapter proteins to facilitate ER-to-Golgi transport and proteolytic activation of SREBPs. When ER cholesterol is above a threshold concentration, this transport is blocked, a process that requires Insigs. However, the precise molecular mechanisms by which cholesterol and Insigs control the conformations of Scap remain unknown. Here, we elucidate the 3.2 Å cryo-EM structure of a Scap/Insig complex in the presence of saturating amounts of cholesterol. Structure-guided mutagenesis of the Scap/Insig interface indicates that Scap’s transmembrane helix 7 (TM7) plays a critical role in transducing conformational changes between the luminal and cytosolic sides of the ER membrane to control Scap/SREBP transport from ER to Golgi. An intramembrane cholesterol bound at the Scap/Insig interface competes with the intramolecular interaction of Scap’s TM7 at this interface to modulate cholesterol sensing. These results further advance our understanding of how Scap senses cholesterol and provides additional targets for controlling cholesterol and lipid synthesis in the context of metabolic diseases and even some cancers.

## Significance

Cholesterol levels in animal cells are controlled by a homeostatic feedback mechanism that regulates activation of sterol regulatory element–binding proteins (SREBPs) via the interaction of the membrane proteins Scap and Insig. Our knowledge of how cholesterol modulates the Scap–Insig interaction and SREBP feedback remains elusive. Here, we provide structural and functional evidence that a membrane-embedded cholesterol displaces a transmembrane helix (TM7) on Scap’s Insig-binding surface and glues together the Scap–Insig complex. Our structure-guided mutagenesis studies show that Scap’s TM7 competes for binding with cholesterol, and its association promotes trafficking and proteolytic activation of SREBPs. These findings uncover an important way in which cholesterol directly modulates SREBP signaling, and provide a molecular target for inhibition of SREBPs in lipid-related diseases.

**Fig. 1.**
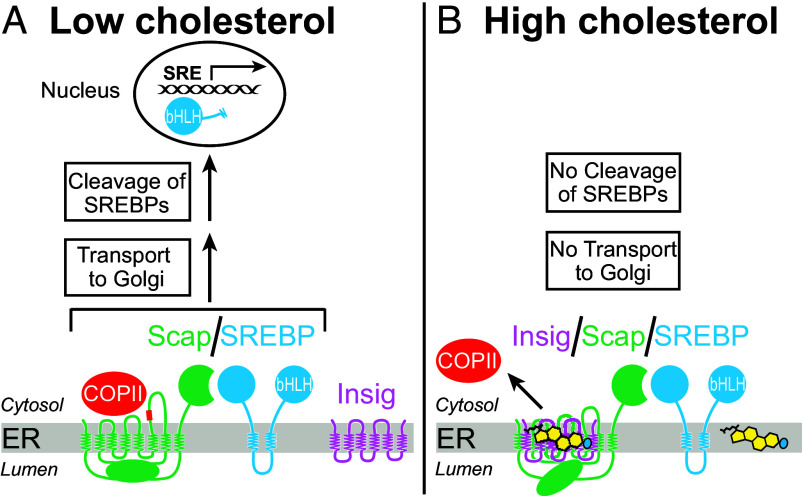
Overview of the Scap/SREBP pathway. (*A*) When cellular cholesterol levels are low, Scap escorts SREBPs from the ER to Golgi, where proteolytic cleavage releases the transcription factor domain of SREBPs for travel to the nucleus and activation of genes involved in lipid production. (*B*) When cholesterol levels rise above a threshold, the sterol binds to Scap and changes Scap’s conformation to promote binding to Insigs. This blocks Scap’s transport to Golgi, which prevents proteolytic cleavage of SREBPs and reduces transcription of genes involved in lipid production. *bHLH*, basic helix-loop-helix-leucine zipper.

The levels of cholesterol, fatty acids, and triglycerides in animal cells are controlled by a regulatory machine in the endoplasmic reticulum (ER) (1, 2). This machine consists of sterol regulatory element–binding proteins (SREBPs), which are membrane-bound transcription factors, and a cholesterol-sensing membrane protein called Scap that binds SREBPs (3). When cholesterol drops below a threshold (4), the Scap/SREBP complexes bind to COPII adapter proteins and are packaged into transport vesicles that shuttle the complexes from the ER to the Golgi, where SREBPs are proteolytically processed to release their active transcription factor domains that then travel to the nucleus to upregulate lipogenic gene transcription (Fig. 1*A*) (5). When cholesterol rises above a threshold concentration, the sterol binds to Scap (6–8), which promotes the binding of Scap to ER retention factors called Insigs (9). In this Insig-bound conformation, the Scap/SREBP complexes do not bind COPII adapter proteins and are not transported to the Golgi (10, 11), thus preventing the proteolytic activation of SREBPs and reducing lipogenic gene transcription (Fig. 1*B*). Oxysterols such as 25-hydroxycholesterol (25HC) bind Insigs and promote the Scap–Insig interaction, which also leads to reduced SREBP activation (12, 13).

More than two decades of cellular, biochemical, and structural studies have led to a working model for how Scap senses changes in ER cholesterol levels (3, 14). Scap is an ~1,300 amino acid membrane protein that binds SREBPs through its cytosolic carboxy-terminal domain (~500 amino acids). The remainder of Scap contains 8 transmembrane (TM) helices, with TMs 2-6 comprising a “sterol-sensing domain” (SSD) (15) that binds Insigs and has structural homology to the cholesterol biosynthetic enzyme HMG-CoA reductase (16), the cholesterol transport proteins NPC1 (17) and NPC1L1 (18), as well as the cholesterol-transporting signaling protein Patched (19, 20). Scap’s 8 TM helices are connected by 7 loops, three of which are large and perform important functional roles. The cytosolic Loop 6 (L6) contains a binding site for COPII adapter proteins (10), and the luminal loops 1 and 7 (L1 and L7) bind to each other (8, 21) and contain a cholesterol-binding site (7). The L1–L7 luminal domain is oriented directly below the SSD when ER cholesterol is low. A rise in ER cholesterol levels leads to binding to Insigs and a large 215° rotation that repositions Scap’s L1–L7 domain under the ER membrane (14). This large rotation changes the conformation of L6 to prevent its binding to COPII proteins thus preventing transport of Scap to the Golgi. It is currently unknown how cholesterol mediates the communication between the L1–L7 domain on the luminal side of the ER and L6 on the cytosolic side.

In this study, we sought to identify the molecular basis for how cholesterol interacts with Scap using cryo-EM structure determination. Here, we report the structure of a Scap/Insig complex that is purified in the presence of saturating amounts of cholesterol. Our Scap/Insig structure, combined with extensive mutagenesis, implicates a key role for TM7, which connects the L1–L7 domain to L6, in modulating Scap’s response to cholesterol. The structure reveals a cholesterol molecule bound at the Scap/Insig interface which competes with TM7, the critical L1–L7–L6 linkage, and acts as a molecular glue to promote complex formation. Together, these findings clarify how Scap senses cholesterol to control the activation of SREBPs and the lipid content of cells.

## Results

### Development of a Hamster Scap Construct for Structural Studies.

Our working model for how Scap functions was established primarily through studies of the hamster ortholog of Scap (3). To test this model with a structural framework, we need structures of hamster Scap. However, our structural studies to date have been on the chicken ortholog of Scap, which is only ~75% identical to hamster Scap. To obtain structures of hamster Scap, we revisited the technical advances that enabled our earlier cryo-EM structural determination of chicken Scap. The key reagent in these studies was a Fab fragment (4G10^Fab^) (14) that bound the L1–L7 domain of chicken Scap and provided a marker for particle alignment during cryo-EM analysis. Unfortunately, 4G10^Fab^ does not bind to hamster Scap (see below). We wondered whether we could modify the L1–L7 domain of hamster Scap so it would be able to bind 4G10^Fab^. A close examination of the binding interface of chicken Scap’s L1–L7 domain to 4G10^Fab^ showed that 33 Scap residues were within 7.5 Å of 4G10^Fab^ (*SI Appendix*, Fig. S1). Of these 33 4G10^Fab^-contacting chicken Scap residues, seven were not conserved in hamster Scap (*SI Appendix*, Fig. S1). We mutated these seven residues on hamster Scap to their chicken counterpart, and the resulting mutant version of hamster Scap is designated as Scap^EM^.

We first checked whether Scap^EM^ could bind 4G10^Fab^. For these initial tests, we purified the L1–L7 domains of both wildtype (WT) hamster Scap (Scap^WT^) and mutant Scap^EM^ (*SI Appendix*, *Methods*). We also generated large amounts of recombinant 4G10^Fab^ from mammalian cells (*SI Appendix*, *Methods* and Fig. S2 *A* and *B*). We then incubated the purified L1–L7 domain of Scap^EM^ with 4G10^Fab^ and subjected the mixture to gel filtration chromatography. For comparison, we also conducted gel filtration analysis of the L1–L7 domain of Scap^EM^ in the absence of 4G10^Fab^. As shown in *SI Appendix,* Fig. S2*C*, the L1–L7 domain of Scap^EM^ eluted as a single peak at ~15 mL (*blue curve in top panel*). In contrast, the elution profile of the mixture of Scap^EM^’s L1–L7 and 4G10^Fab^ showed two peaks consistent with formation of a complex (*SI Appendix,* Fig. S2*C*, *red curve in top panel and gels in bottom panel*). In contrast, the L1–L7 domain of hamster Scap^WT^ did not interact with 4G10^Fab^ (*SI Appendix,* Fig. S2*D*, *blue and red curves in top panel*, *gels in bottom panel*).

**Fig. 2.**
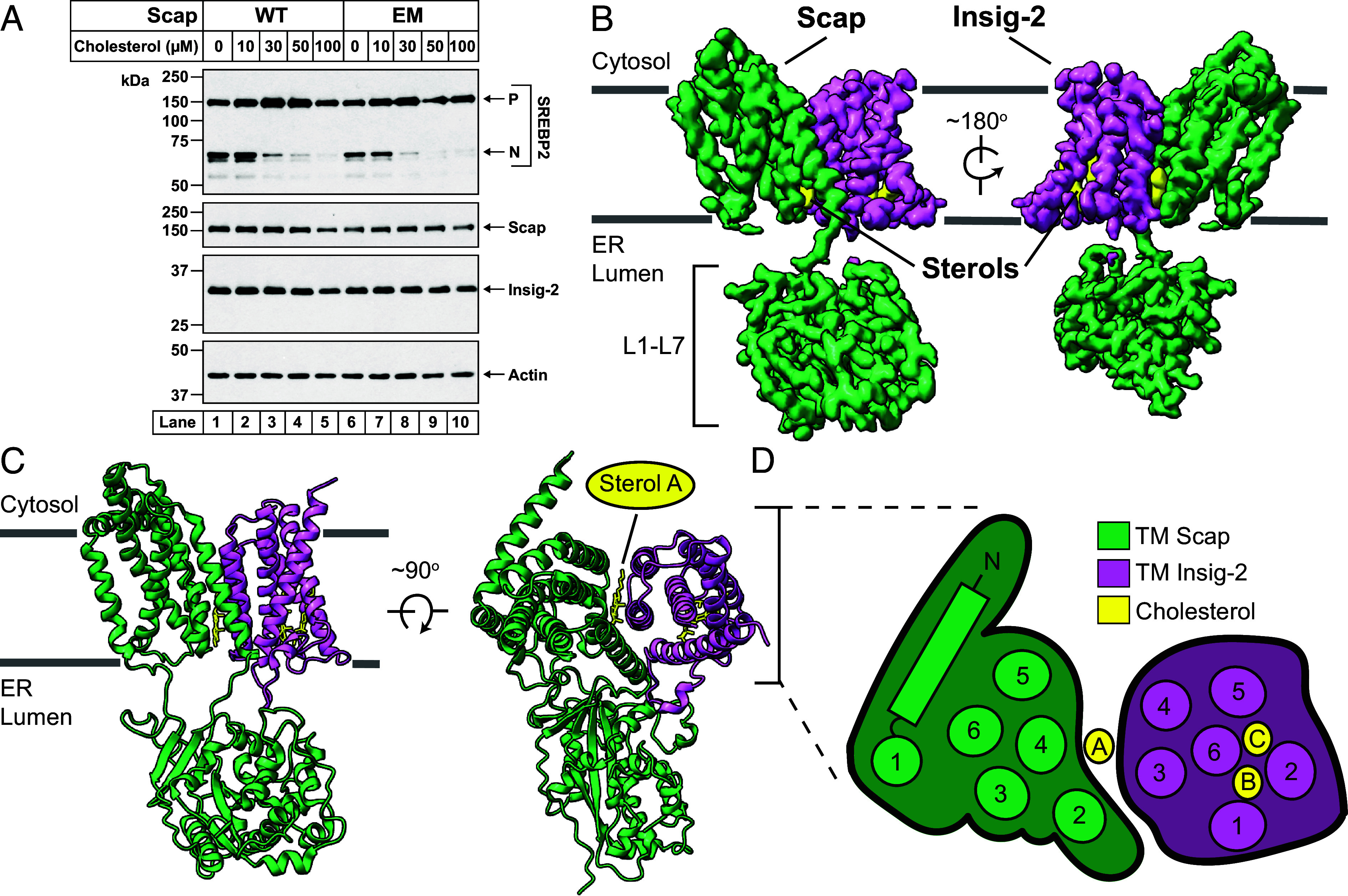
Structure of a Scap/cholesterol/Insig-2 complex. (*A*) Comparison of the ability of WT and mutant (EM) Scap to mediate SREBP2 cleavage. Scap-deficient SRD-13A cells were set up and transfected as described in *SI Appendix*, *Methods* with 2 µg of pTK-3xFLAG-SREBP2, 1.5 µg of pTK-Insig-2-6xMyc, and 2 µg of either pTK-Scap^WT^ or pTK-Scap^EM^. The transfected cells were then depleted of cholesterol and replenished with the indicated concentrations of cholesterol (delivered in complexes with MCD), after which cell lysates were analyzed by immunoblot as described in *SI Appendix*, *Methods*. *P*, precursor form of SREBP2; *N*, cleaved nuclear form of SREBP2. (*B*) Structure of Scap^EM^(TM1-8) bound to Insig-2. Cryo-EM analysis of the cholesterol-mediated Scap^EM^(TM1-8)/Insig-2 complex was carried out as described in *SI Appendix*, *Methods* and the DeepEMhanced reconstruction was contoured to 5σ and colors were assigned by docking the de novo model of Scap/Insig-2 with cholesterol using colorzone in ChimeraX. (*Left*) Scap^EM^(TM1-8) is colored green, Insig-2 is colored magenta, and extra densities modeled as cholesterol are colored yellow. (*Right*) The reconstruction was rotated ~180° relative to the *Left* panel. (*C*) Model of Scap^EM^(TM1-8) bound to Insig-2. The de novo model of Scap/Insig with three modeled cholesterols (*SI Appendix*, *Methods*) is shown in the same position as in (*B*). One of the modeled cholesterol molecules is termed sterol A. (*D*) Visualization of cholesterol bound to the Scap^EM^(TM1-8)/Insig-2 complex. (*Left*) The model of the Scap/Insig complex with cholesterol is shown in the same position as in (*C*) but rotated ~90° relative to the membrane and viewed from the cytosolic side. (*Right*) An enlarged model outline of the cytosolic membrane view of the Scap/Insig complex showing the relative arrangement of transmembrane helices (numbered). The three modeled cholesterols are represented by yellow ovals (lettered).

Having verified that the seven mutations in hamster Scap^EM^ allowed binding to 4G10^Fab^, we next checked whether these mutations affected its cellular function in an assay that we routinely use to assess Scap constructs. In this assay, Scap-deficient Chinese hamster ovary (CHO) cells (designated as SRD-13A) (22) were transfected with either Scap^WT^ or Scap^EM^, along with the human orthologs of SREBP2, one of the three isoforms of SREBP, and Insig-2, one of the two isoforms of Insig. When the Scap^WT^-transfected cells were depleted of cholesterol, we detected a large amount of the cleaved nuclear form of SREBP2, indicating that Scap^WT^ had transported SREBP2 to the Golgi for processing (Fig. 2*A*, *lane 1*). When these sterol-depleted cells were replenished with cholesterol (delivered in complexes with methyl-β-cyclodextrin (MCD)), we observed a gradual decline in the cleaved form of SREBP2 (Fig. 2*A*, *lanes 2*–*5*), consistent with the notion that the delivered cholesterol bound to Scap^WT^ and prevented Scap^WT^’s transport to Golgi. An identical result was observed with Scap^EM^ (compare *lanes 6*–*10* to *lanes 1*–*5*), indicating that Scap^EM^ responded to cholesterol in a similar fashion as Scap^WT^. Having thus confirmed the functionality of Scap^EM^, we embarked on structural studies of this version of hamster Scap.

### Structure of a Cholesterol-Bound Scap/Insig Complex.

For structural analysis, we generated a plasmid encoding a dual-tagged version of Scap^EM^ with a His_8_-tag at the NH_2_-terminus and a 3xFLAG tag at the COOH terminus. We overexpressed tagged Scap^EM^ in HEK293 GnTI^-^ cells, solubilized the membranes in lauryl maltose neopentyl glycol (LMNG) detergent, and purified Scap^EM^ by FLAG affinity chromatography (*SI Appendix*, *Methods*). The purified protein was then exchanged into glycol-diosgenin (GDN) detergent and incubated with 4G10^Fab^ (*SI Appendix,* Fig. S3*A*). Cryo-EM analysis of the resulting Scap^EM^/4G10^Fab^ complex (*SI Appendix,* Fig. S3 *B* and *C*) showed high local resolution (2.8 Å) for the L1–L7 domain (*SI Appendix,* Fig. S3*D* and Table S1), but the membranous portion of Scap^EM^ was not resolved with only the CTD observed as a protrusion from the detergent micelle (*SI Appendix,* Fig. S4*A*). Even with these limitations, we could ascertain that the L1–L7 domain was under Scap^EM^’s membrane domain, similar to what had been observed in our previous structure of chicken Scap (*SI Appendix,* Fig. S4 *A* and *B*) (14).

We proceeded to study complexes of Scap^EM^ with Insig in the presence of cholesterol. For these studies, we generated two plasmids. One plasmid encoded a dual-tagged version of Scap^EM^ (3xFLAG and His_8_ tags at the NH_2_ terminus) that included the entire membrane-embedded portion (TM1-8) but lacked the SREBP-interacting COOH-terminal domain. This version of Scap^EM^ is hereafter referred to as Scap^EM^(TM1-8). The other plasmid encoded full-length hamster Insig-2 with no affinity tags. We overexpressed Scap^EM^(TM1-8) along with hamster Insig-2 in HEK293 GnTI^-^ cells grown in lipoprotein-rich serum containing high levels of cholesterol, and after membrane solubilization in LMNG, we purified the Scap^EM^(TM1-8)/Insig-2 complex by FLAG affinity chromatography. The purified protein complex was then exchanged into GDN detergent saturated with cholesterol (~10 µM) (*SI Appendix*, *Methods*), after which we incubated the complex with 4G10^Fab^. The resulting monodisperse sample contained a stoichiometric complex of the three proteins (*SI Appendix,* Fig. S5*A*) and was used for single-particle cryo-EM analysis. After multiple rounds of 2D and 3D classification of a large (17-million particle) dataset, we obtained a reconstruction of the cholesterol-stabilized Scap^EM^(TM1-8)/Insig-2/4G10^Fab^ complex at an overall resolution of 3.2 Å (*SI Appendix,* Fig. S5 *B*–*E* and Table S1). The resulting cryo-EM map was mostly uniform throughout the transmembrane and L1–L7 domains of Scap and the TMs of Insig-2, representing the most complete map of this entire complex (Fig. 2*B*).

When comparing this Scap^EM^(TM1-8)/Insig-2/4G10^Fab^ structure to that of Scap^EM^/4G10^Fab^, we observed a striking difference in the orientation of the L1–L7 domains. While L1–L7 is oriented under the membrane domain in the Scap^EM^/4G10^Fab^ structure, L1–L7’s positioning is rotated away from Scap^EM^’s membrane domain in the Scap^EM^(TM1-8)/Insig-2/4G10^Fab^ structure (*SI Appendix,* Fig. S6 *A* and *B*). This rotation is similar to what we had previously observed when comparing structures of chicken Scap alone to that of chicken Scap in complex with chicken Insig-1 (14). The previous studies of chicken Scap/Insig complexes were carried out without any added cholesterol and with a mutant version of Scap that bound Insig in the absence of cholesterol (23). In contrast, our current structures used a version of Scap that did not contain any such mutations that promoted constitutive binding to Insigs, but rather relied on cholesterol to drive the Scap–Insig interaction. We were thus hopeful that our structures could provide insights into how cholesterol changed Scap’s conformation to promote binding to Insigs.

Our reconstruction of Scap^EM^(TM1-8)/Insig-2/4G10^Fab^ permitted building of a de novo atomic model and revealed three sterol-shaped densities in the transmembrane region (Fig. 2 *B* and *C* and *SI Appendix,* Fig. S6 *C* and *D*). Inasmuch as we had saturated the samples with cholesterol, we modeled these densities as cholesterol. One of the densities, at the Scap^EM^/Insig-2 interface (sterol A in Fig. 2*D*), is at a similar position where 25HC, an oxysterol that binds Insigs but not Scap (13), and digitonin (a detergent that contains the steroid nucleus) were modeled in previous structures of human Scap/Insig-2 (24, 25) (*SI Appendix*, Fig. S6*E*). The other two densities, sterols B and C, were embedded within Insig-2 (Fig. 2*D*). Notably, none of the three densities were fully contained within Scap^EM^(TM1-8). Since our goal is to understand how cholesterol controls Scap’s structure and function, we focused on the one sterol density that is in contact with Scap, namely sterol A (Fig. 2*D* and *SI Appendix*, Fig. S6*C*).

### Mutational Analysis of Scap Residues at the Scap/Cholesterol/Insig Interface.

**Fig. 3.**
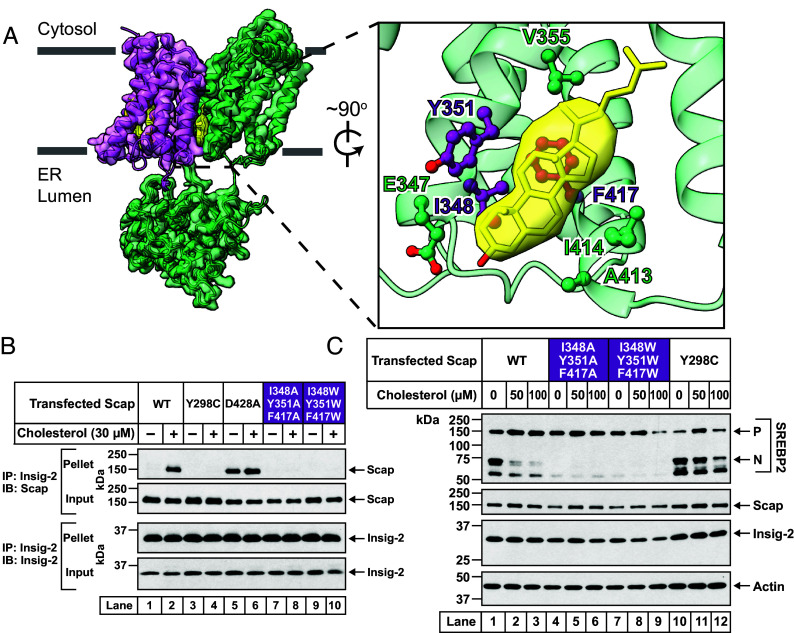
Identification of a new class of Scap mutations that disrupt its interaction with Insig but still prevent transport to Golgi. (*A*) Structural model. Shown is the model and cryo-EM reconstruction of Scap^EM^(TM1-8) (*green*) bound to Insig-2 (*magenta*) with associated sterols (*yellow*) (similar orientation as Fig. 2 *B*, *Right* panel). (*Inset*) A zoomed-in view of the region where Scap and cholesterol (sterol A) interact. Sidechains of the seven Scap residues that were within 5Å of cholesterol (sterol A) are shown as ball and stick models. Three of these seven residues (I348, Y351, F417) that were subjected to mutational analysis in the following panels are colored purple and the remaining four residues are colored green. (*B* and *C*) Comparison of functional properties of WT and triple mutant versions of Scap. (*B*) Binding to Insigs. Scap-deficient SRD-13A cells were set up and transfected as described in *SI Appendix*, *Methods* with 2 μg of pTK-Insig-2-6xMyc and 4 µg of either WT or indicated mutant versions of pTK-Scap. The transfected cells were then depleted of cholesterol and replenished with the indicated concentrations of cholesterol (delivered in complexes with MCD), after which the cells were harvested and processed for coimmunoprecipitation assays and immunoblot analysis as described in *SI Appendix*, *Methods*. (*C*) Mediating cleavage of SREBP2. Scap-deficient SRD-13A cells were set up and transfected as described in *SI Appendix*, *Materials and Methods* with 2 µg of pTK-3xFLAG-SREBP2, 1.5 µg of pTK-Insig-2-6xMyc, and 2 µg of either WT or indicated mutant versions of pTK-Scap. The transfected cells were then depleted of cholesterol and replenished with the indicated concentrations of cholesterol (delivered in complexes with MCD), after which cell lysates were analyzed by immunoblot as described in *SI Appendix*, *Methods*. *P*, precursor form of SREBP2; *N*, cleaved nuclear form of SREBP2.

A thorough examination of the vicinity of sterol A revealed contacts with twelve residues, seven on Scap (E347, I348, Y351, V355, A413, I414, F417) and five on Insig-2 (I118, H120, A121, K124, L140). We first focused on Scap, and particularly on residues I348, Y351, and F417 in closest contact with sterol A (Fig. 3*A*). We reasoned that mutation of each of these large hydrophobic residues to a smaller residue such as alanine could remove important packing contacts at the sterol A pocket whereas mutation to a larger residue such as tryptophan could fill the pocket, both leading to a potential disruption of the sterol interaction. To test this idea, we prepared two triple mutant versions of hamster Scap, hereafter designated as Scap^I348A/Y351A/F417A^ and Scap^I348W/Y351W/F417W^. We purified His_8_-tagged versions of both mutant Scaps as well as WT Scap in LMNG detergent (*SI Appendix*, *Methods*). The purified mutant Scaps exhibited similar gel filtration chromatography profiles as WT Scap (*SI Appendix*, Fig. S7*A*), suggesting that the disruptive mutations did not grossly affect the protein. We then assessed the binding of cholesterol to these mutant Scaps using a previously developed assay(14). Both mutant Scaps bound [^3^H]cholesterol in a similar fashion as WT Scap (*SI Appendix*, Fig. S7*B*). We were not entirely surprised by this result because the previously developed sterol-binding assay reports on a binding site in Scap’s L1–L7 domain that shows all-or-none specificity for cholesterol over 25HC (8, 26). In contrast, the observed sterol A site is between Scap and Insig-2, not in the L1–L7 domain, and can accommodate cholesterol as well as 25HC. The lack of an effect of these mutations in this binding assay may also reflect that Insig-2 likely contributes in a cooperative fashion to the interfacial sterol affinity, and our assay probes the binding of [^3^H]cholesterol to Scap alone. To understand the importance of the sterol A site, we used two other assays of Scap function, namely its cholesterol-induced interaction with Insigs and the cholesterol-induced block of its transport from ER to Golgi.

First, we performed a coimmunoprecipitation assay to assess the interaction of Scap with Insig. We transfected Scap-deficient SRD-13A cells with plasmids encoding Myc-tagged Insig-2 and WT or the above-described triple mutant versions of Scap. The transfected cells were first treated with 2-hydroxypropyl-β-cyclodextrin (HPCD) to lower cholesterol levels, after which the cells were incubated with cholesterol/MCD complexes to raise cholesterol levels. The cell membranes were then solubilized and the Myc-tagged Insig-2 was isolated using anti-Myc agarose beads. When cholesterol was low, very little WT Scap was detected in the eluate and this increased as cholesterol was added to the cells (*lanes 1 and 2*, Fig. 3*B*). As controls, we tested two previously described mutations of Scap. One mutation, Y298C, fails to bind Insig-2 even in the presence of cholesterol (*lanes 3 and 4*, Fig. 3*B*) (27), while the other mutation, D428A, constitutively binds Insig-2 even in the absence of cholesterol (*lanes 5 and 6*, Fig. 3*B*) (23, 28). Both Scap^I348A/Y351A/F417A^ and Scap^I348W/Y351W/F417W^ failed to bind Insig-2, even when the cells were treated with cholesterol (*lanes 7*–*10*, Fig. 3*B*). In this aspect, the triple mutants of Scap behave similarly to the Y298C mutant version of Scap. If this similarity were to hold, then we would expect that the inability of the triple mutants to bind Insig would lead to uninhibited transport of Scap from ER to Golgi during cholesterol-replete conditions, leading to constitutive cleavage of SREBPs.

We tested this idea with the SREBP cleavage assay described in Fig. 2*A*. As expected, cleavage of SREBP2 declined with cholesterol treatment when the cells were transfected with WT Scap, but remained unchanged when transfected with the Y298C mutant version of Scap (*compare lanes 1*–*3 with 10–12*, Fig. 3*C*). Surprisingly, no cleavage was detected when the cells were transfected with either Scap^I348A/Y351A/F417A^ (*lanes 4*–*6*, Fig. 3*C*) or Scap^I348W/Y351W/F417W^ (*lanes 7*–*9*, Fig. 3*C*). Nevertheless, the inability of Scap^I348A/Y351A/F417A^ or Scap^I348W/Y351W/F417W^ to transport SREBP2 to the Golgi was unlikely to be caused by gross alterations of the structure of these mutant Scaps, since they were both functional in binding to cholesterol (*SI Appendix*, Fig. S7*B*). Thus, these triple mutants represent a new type of Scap mutant that fail to bind Insigs (like Y298C, but unlike D428A) and fail to transport to the Golgi (unlike Y298C, but like D428A).

**Fig. 4.**
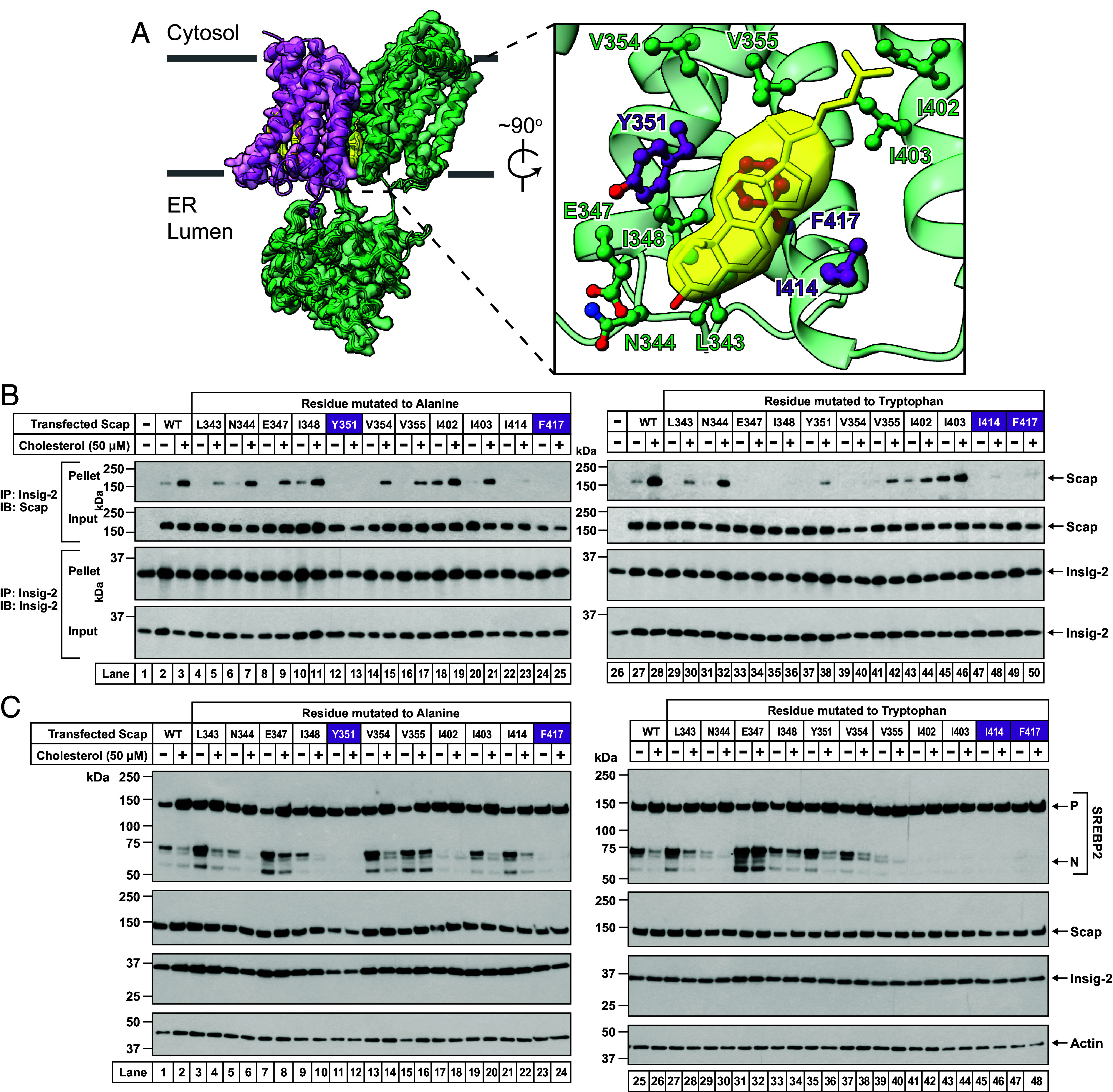
Mutational analysis of Scap residues at the Scap/cholesterol/Insig-2 interface. (*A*) Structural model. Shown is the model and cryo-EM reconstruction of Scap^EM^(TM1-8) (*green*) bound to Insig-2 (*magenta*) with associated sterols (*yellow*) (same as in Fig. 3 *A*, *Left panel*). (*Inset*) A zoomed-in view of the region where Scap and cholesterol (sterol A) interact. Sidechains of eleven Scap residues that were in close proximity of cholesterol (sterol A) are shown as ball and stick models. Three of these eleven residues (Y351, I414, F417) are colored purple, the remaining eight residues are colored green. Each of these 11 Scap residues were mutated to either alanine or tryptophan. (*B*) Binding of Scap mutants to Insigs. Scap-deficient SRD-13A cells were set up and transfected as described in *SI Appendix*, *Methods* with 2 μg of pTK-Insig-2-6xMyc and 4 µg of either WT or indicated mutant versions of pTK-Scap. The control conditions in *lanes 1* and *26* were transfected with 2 μg of pTK-Insig-2-6xMyc and 4 µg of pcDNA3 empty vector plasmid. The transfected cells were then depleted of cholesterol and replenished with the indicated concentrations of cholesterol (delivered in complexes with MCD), after which the cells were harvested and processed for coimmunoprecipitation assays and immunoblot analysis as described in *SI Appendix*, *Methods*. (*C*) Ability of Scap mutants to mediate cleavage of SREBP2. Scap-deficient SRD-13A cells were set up and transfected as described in *SI Appendix*, *Methods* with 2 µg of pTK-3xFLAG-SREBP2, 1.5 µg of pTK-Insig-2-6xMyc, and 2 µg of either WT or indicated mutant versions of pTK-Scap. The transfected cells were then depleted of cholesterol and replenished with the indicated concentrations of cholesterol (delivered in complexes with MCD), after which cell lysates were analyzed by immunoblot as described in *SI Appendix*, *Methods*. *P*, precursor form of SREBP2; *N*, cleaved nuclear form of SREBP2. (*B* and *C*) Mutations shaded in purple (Y351A, I414W, F417A, and F417W) represent additional examples of the new class of Scap mutations described in Fig. 3 that disrupt Scap’s interaction with Insig but still prevent Scap’s transport to Golgi.

To further characterize what this new type of Scap mutant could tell us about Scap’s function, we made single mutations to either alanine or tryptophan of each of I348, Y351, and F417, as well as eight other Scap residues that were within close proximity of sterol A (Fig. 4*A*). We then assessed the ability of these 22 Scap mutants to bind Insig using the assay described above, and the results are shown in Fig. 4*B*. Mutation of I348 to alanine did not disrupt the ability of Scap to bind Insig-2 in a cholesterol-dependent fashion (*compare lanes 10*–*11 to lanes 2*–*3*), whereas mutation to tryptophan abolished this interaction (*compare lanes 35*–*36 to lanes 27*–*28*). In contrast, mutation of Y351 or F417 to either alanine or tryptophan disrupted Scap’s ability to bind Insig-2 in the presence of cholesterol (*lanes 12*–*13*, *24*–*25*, *37*–*38*, *49*–*50*). Additionally, mutation of I414 to either alanine (*lanes 22*–*23*) or tryptophan (*lanes 47*–*48*), or mutation of E347 or V354 to tryptophan (*lanes 33*–*34*, *39*–*40*) also disrupted the Scap–Insig-2 binding. In contrast, mutation of I402 to alanine or I403 to tryptophan promoted Scap’s binding to Insig-2 in the absence of cholesterol (*lanes 18*, *45*). The remaining 10 Scap mutants bound Insig-2 in a similar cholesterol-dependent fashion as WT Scap (*all other lanes*).

We then tested these 22 Scap mutants in assays measuring Scap transport from ER to Golgi, as judged by SREBP2 cleavage (Fig. 4*C*). Of the eleven Scap mutants that displayed alterations in cholesterol-dependent binding to Insig-2, three of them (Y351W, V354W, and I414A) did not significantly alter SREBP2 cleavage (*compare lanes 35*–*36*, *37*–*38*, *21*–*22 to lanes 1*–*2 and 25*–*26*), suggesting that these mutations were not severe enough to affect Scap transport. Two of the remaining eight mutants, E347W and I348W, that were unable to bind Insig-2 (Fig. 4*B*), transported to the Golgi even in the presence of cholesterol (*lanes 32*, *34*, Fig. 4*C*). These two mutants are similar to the previously described Y298C mutation that does not bind Insigs and continues to constitutively transport to the Golgi. Of the remaining six mutants, I402A and I403W, that bound Insig-2 in the absence of cholesterol, failed to transport to the Golgi even in cholesterol-depleted conditions (*lanes 17*–*18*, *43*–*44*, Fig. 4*C*). These two mutants are similar to the previously described D428A mutation that constitutively binds Insigs and does not transport to the Golgi regardless of the membrane cholesterol content. The remaining four mutants, Y351A, I414W, F417A, and F417W, are similar to the triple mutants described above (Fig. 3) in that they do not bind Insigs and do not transport to Golgi (*lanes 11*–*12*, *23*–*24*, *45*–*48*, Fig. 4*C*). These four point mutants (shaded purple), as with the triple mutations above, represent a new class of Scap mutations that lack both Insig binding and transport to Golgi. The three residues thus identified are at the center of the sterol A pocket and make the most intimate contacts with the sterol nucleus (Figs. 3*A* and 4*A*).

### Mutational Analysis of Insig Residues at the Scap/Cholesterol/Insig Interface.

We next focused on the Insig side of the Scap/sterol/Insig interface, and in particular on the five Insig-2 residues (I118, H120, A121, K124, L140) that contact sterol A (*SI Appendix*, Fig. S8*A*). We made single mutations of each of these residues to either alanine or tryptophan, with the exception of A121 which was only mutated to tryptophan. We also did not consider mutation of L140 to tryptophan. We then used SRD-15 cells (29), which lack both Insig-1 and Insig-2, to test the ability of each of these eight Insig-2 mutants to bind Scap. After transfection with plasmids encoding WT or mutant versions of Myc-tagged Insig-2 along with HA-tagged WT Scap, we depleted the cells of sterols and then incubated the sterol-depleted cells with cholesterol/cyclodextrin complexes. Cell membranes were solubilized and the Myc-tagged Insig-2 was isolated using anti-Myc agarose beads. In the case of transfection with WT Insig-2, we detected little amounts of Scap that coimmunoprecipitated with Insig-2 in the absence of sterols, and this amount increased upon cholesterol addition (*lanes 3*–*4*, *SI Appendix*, Fig. S8*B*). Two of the eight mutant versions of Insig-2 (H120A, K124A) also bound Scap upon cholesterol addition in a manner similar to WT Insig-2 (*compare lanes 7*–*10 to lanes 3*–*4*, *SI Appendix*, Fig. S8*B*). In contrast, the other six mutant versions of Insig-2 (I118A, L140A, I118W, H120W, A121W, K124W) did not bind Scap in the presence of cholesterol (*lanes 5*–*6*, *11*–*12*, *17*–*24*, *SI Appendix*, Fig. S8*B*). We then tested these eight Insig mutants that were deficient in binding Scap in assays measuring Scap transport to Golgi, as judged by SREBP2 cleavage (*SI Appendix*, Fig. S8*C*). The two mutant versions of Insig-2 that bound Scap in a cholesterol-regulated manner (H120A, K124A) also regulated Scap’s transport to Golgi and subsequent SREBP2 cleavage in a manner similar to WT Insig-2 (*compare lanes 5*–*8 with lanes 1*–*2*, *SI Appendix*, Fig. S8*C*). Two of the other six mutant versions of Insig-2 that did not coimmunoprecipitate Scap (I118A, L140A; *lanes 3*–*4*, *9*–*10*, *SI Appendix*, Fig. S8*B*) retained the ability to regulate Scap’s transport to Golgi similarly to WT Insig-2, suggesting that these mutations were not severe enough to alter Scap transport. One mutant, I118W, showed reduced expression (*lanes 13*–*14*, *SI Appendix*, Fig. S8*C*) and was excluded from further analysis. The remaining three mutant versions of Insig-2 (H120W, A121W, K124W) that did not bind Scap also did not block Scap transport and SREBP2 cleavage in the presence of cholesterol (*lanes 15*–*20*, *SI Appendix*, Fig. S8*C*).

The above analysis of 30 mutations at the Scap/sterol A/Insig interface reveals a diverse range of alterations to the Scap/Insig/SREBP pathway (*SI Appendix*, Tables S2 and S3). Two Scap mutations promote formation of the Scap/Insig complex without cholesterol, thus disrupting Scap transport and SREBP cleavage even in the absence of added cholesterol. Other mutations (two in Scap, three in Insig-2) have the opposite effect. They disrupt the cholesterol-induced Scap–Insig interaction, leading to constitutive Scap transport and SREBP cleavage even in the presence of added cholesterol. Four Scap mutations (to three residues) also disrupt the cholesterol-induced Scap–Insig interaction, but these mutants do not transport to Golgi, leading to a block of SREBP cleavage even in low cholesterol. Thus, this interfacial region is a hotspot for modulation of the Scap–Insig interaction and Scap transport from ER to Golgi. All the Scap residues in this hotspot whose mutation alters cholesterol regulation of the SREBP pathway are localized to Scap’s TM4 (E347, I348, Y351), TM5 (I402, I403), or TM6 (I414, F417). Therefore, we sought to understand the cholesterol-mediated changes at the surface formed by these three Scap helices.

### Identification of a Regulatory Role for Scap’s TM7.

**Fig. 5.**
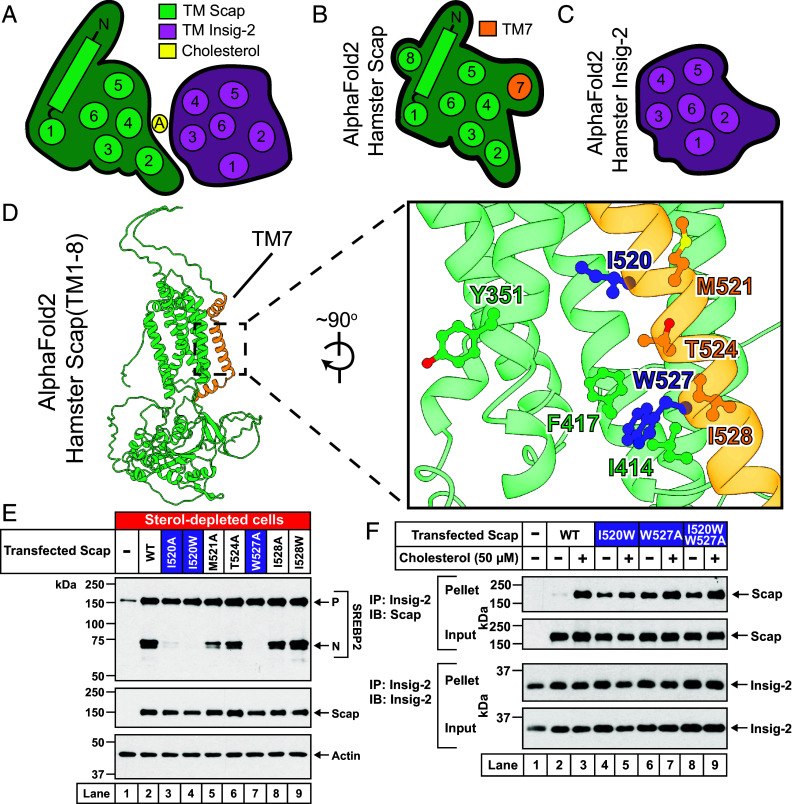
Competition between Insig-2 and Scap’s TM7 for a common binding site on Scap. (*A*–*C*) Comparison of the structure of the Scap/Insig-2 complex to AlphaFold2 prediction models of Scap and Insig-2 alone. (*A*) Scap/Insig-2 complex. Shown is a model outline of the cytosolic membrane view of the Scap/Insig-2 complex with the relative arrangement of transmembrane helices of Scap (*green*) and Insig-2 (*magenta*). Sterol A is shown in yellow. (*B*) Scap. Shown is a model outline of the cytosolic membrane view of hamster Scap as predicted by AlphaFold2 [AF-P97260-F1-v4] with the relative arrangement of transmembrane helices (TM7 in *orange*, all other TMs in *green*). (*C*) Insig-2. Shown is a model outline of the cytosolic membrane view of hamster Insig-2 as predicted by AlphaFold2 [AF-Q8CFA5-F1-v4] with the relative arrangement of transmembrane helices (*magenta*). (*D*) Predicted structural model. Shown is a cartoon model predicted by AlphaFold2 of the membrane domain of hamster Scap (aa 1-752) [AF-P97260-F1-v4] with TM7 (*orange*) packed against a surface formed by TM4/TM5/TM6 (*green*). (*Inset*) Zoomed-in view of the region of Scap where TM7 interacts with the surface formed by TM4/TM5/TM6. Five residues on TM7 are highlighted, the ones facing the transmembrane surface of Scap are shown in dark blue (I520, W527) whereas the others are shown in orange. As a reference, key residues on the transmembrane surface of Scap that have important functional roles (Fig. 4) are shown in green (Y351, I414, F417). (*E*) Ability of Scap mutants to mediate cleavage of SREBP2. Scap-deficient SRD-13A cells were set up and transfected as described in *SI Appendix*, *Methods* with 2 µg of pTK-3xFLAG-SREBP2 and 2 µg of either WT or indicated mutant versions of pTK-Scap. The control in *lane 1* with no Scap was transfected with 2 μg of pTK-3xFLAG-SREBP2 and 2 µg of pcDNA3 empty vector plasmid. The transfected cells were then depleted of cholesterol, after which cell lysates were analyzed by immunoblot as described in *SI Appendix*, *Methods*. *P*, precursor form of SREBP2; *N*, cleaved nuclear form of SREBP2. (*F*) Binding of Scap mutants to Insigs. Scap-deficient SRD-13A cells were set up and transfected as described in *SI Appendix*, *Methods* with 2 μg of pTK-Insig-2-6xMyc and 4 µg of either WT or indicated mutant versions of pTK-Scap. The control in *lane 1* with no Scap was transfected with 2 μg of pTK-Insig-2-6xMyc and 4 µg of pcDNA3 empty vector plasmid. The transfected cells were then depleted of cholesterol and replenished with the indicated concentrations of cholesterol (delivered in complexes with MCD), after which the cells were harvested and processed for coimmunoprecipitation assays and immunoblot analysis as described in *SI Appendix*, *Methods*.

A clue to the nature of these changes emerged from a comparison of the cholesterol-induced Scap/Insig structure (Fig. 5*A*) to the individual structures of hamster Scap (Fig. 5*B*) and hamster Insig-2 (Fig. 5*C*) predicted by AlphaFold2 (30). The predicted structure of Insig-2 is not significantly altered compared to its structure in the cryo-EM complex with Scap. On the other hand, the predicted structure of hamster Scap shows that TM7 (Fig. 5*B*, *shaded orange*, and *SI Appendix*, Fig. S9*A*) packs against TM4/TM5/TM6 and overlaps with the site of sterol A and Insig binding (Fig. 5 *A* and *B*). When we reexamined our prior structure of chicken Scap (without Insig) (14), we noted that a TM helix with low-resolution density that we had tentatively assigned as TM8 is positioned in close agreement with where TM7 is predicted to be located by AlphaFold2 (*SI Appendix*, Fig. S9*B*). Based on this prediction and evidence below, we believe that our previous low-resolution TM density likely represented TM7 of chicken Scap. Furthermore, in the structure of HMG-CoA reductase (HMGCR), another ER membrane protein whose membrane domain shares similarities with that of Scap, the equivalent TM7 is positioned at a similar overall site relative to its TM4/TM5/TM6 surface (*SI Appendix*, Fig. S9*C*) (16). Based on these analyses, we speculate that, in the absence of cholesterol and Insigs, TM7 in Scap is packed against the TM4/TM5/TM6 surface. In contrast, when Scap is bound to cholesterol and Insigs, TM7 is not resolved (Fig. 2*D*). Previous assignments of TM7 in a different position in structures of the Scap/Insig complex in the presence of 25HC (24, 25) were also speculative. This comparative analysis suggests that TM7 of Scap competes with sterols and Insig for the TM4/TM5/TM6 surface of Scap.

If this was the case, disruptions of TM7 could dissociate this helix from the TM4/TM5/TM6 surface even in low cholesterol, leading to aberrant binding of Scap to Insig, which would block Scap’s transport and subsequent SREBP cleavage. To test this hypothesis, we examined the AlphaFold2-predicted Scap structure and selected five residues on Scap’s TM7 for mutational analysis (*Inset*, Fig. 5*D*). The sidechains of two of these residues, I520 and W527, faced the TM4/TM5/TM6 surface of Scap, whereas the sidechains of the other three residues, M521, T524, and I528, faced the opposite direction. We mutated these residues to alanine or tryptophan and transfected these mutant Scaps into Scap-deficient cells along with SREBP2. After transfection, the cells were depleted of cholesterol, after which we assessed SREBP2 cleavage. As expected, no cleavage was observed in the absence of Scap, whereas WT Scap generated a large amount of cleaved SREBP2 (*lanes 1*, *2*, Fig. 5*E*). Mutation of the TM4/TM5/TM6-facing I520 and W527 blocked SREBP2 cleavage under these sterol-depleted conditions (*lanes 3*, *4*, *7*, Fig. 5*E*). In contrast, mutation of M521, T524, and I528, which do not face the TM4/TM5/TM6 surface, did not affect SREBP2 cleavage (*lanes 5*, *6*, *8*, *9*, Fig. 5*E*). We then focused on the mutants that blocked SREBP2 cleavage and checked whether these mutant Scaps bound Insig-2 in the absence of added cholesterol. The coimmunoprecipitation assay described above shows that I520W, W527A, and the double mutant I520W/W527A all bound Insig-2 both in the absence and presence of added cholesterol (*lanes 4*–*9*, Fig. 5*F*). In contrast, WT Scap bound Insig-2 only in the presence of cholesterol (*lanes 2*, *3*, Fig. 5*F*). We did not examine I520A, which also blocked SREBP2 cleavage (*lane 3*, Fig. 5*E*), in this assay. These results are consistent with a model where the packing of Scap’s TM7 against its TM4/TM5/TM6 surface prevents sterol-induced binding of Insigs to this same surface.

### Locking TM7 to Scap by Disulfide Engineering.

**Fig. 6.**
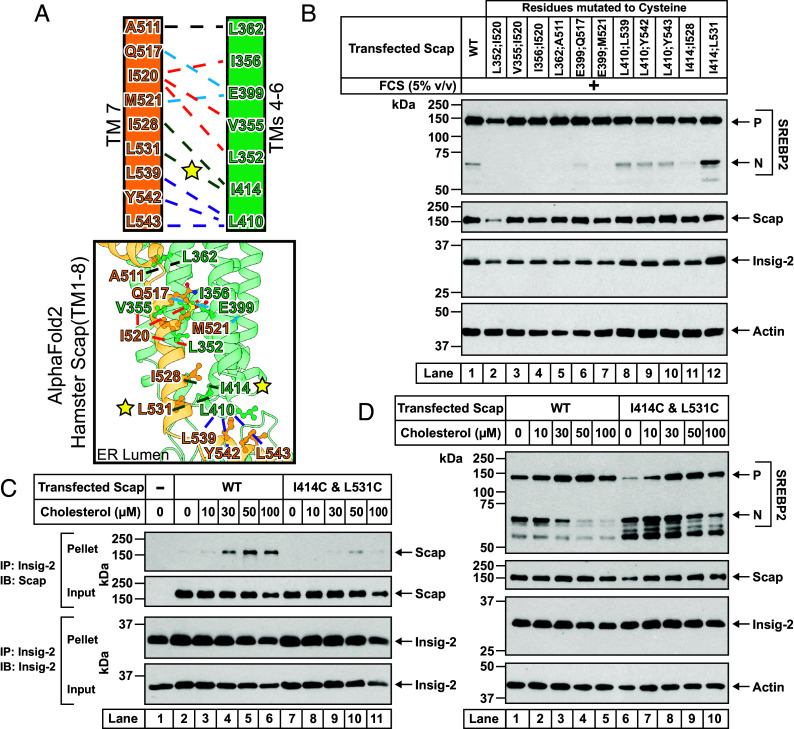
Locking Scap’s TM7 against Scap’s transmembrane surface prevents Scap–Insig binding leading to unregulated transport of Scap to Golgi. (*A*) Schematic illustrating pairs of Scap residues (connected by dotted lines), one on TM7 and the other on the surface formed by TM4/TM5/TM6, that were both mutated to cysteine (*Top panel*). Owing to the close proximity of the pairs of residues, as judged by the AlphaFold2 model of hamster Scap [AF-P97260-F1-v4] (*Bottom panel*) where TM7 is shown in orange and the surface formed by TM4/TM5/TM6 is shown in green, we speculated that the introduced cysteines may form a disulfide bond (dashed lines). Designed pairs that share a residue are grouped by colored dotted lines. The starred colored line (*Top panel*) and starred residues (*Bottom panel*) indicate a successful disulfide pairing as assessed in (*B*–*D*). (*B*) Ability of Scap mutants to mediate cleavage of SREBP2. Scap-deficient SRD-13A cells were set up and transfected as described in *SI Appendix*, *Methods* with 2 µg of pTK-3xFLAG-SREBP2, 1.5 μg pTK-Insig-2-6xMyc, and 2 µg of either WT or indicated double mutant versions of pTK-Scap. The transfected cells were incubated in fresh FCS-rich medium A for 4 h, after which cell lysates were analyzed by immunoblot as described in *SI Appendix*, *Methods*. *P*, precursor form of SREBP2; *N*, cleaved nuclear form of SREBP2. (*C*) Comparison of the ability of WT and mutant Scap (I414C/L531C) to bind to Insigs. Scap-deficient SRD-13A cells were set up and transfected as described in *SI Appendix*, *Methods* with 2 μg of pTK-Insig-2-6xMyc and 4 µg of either WT or pTK-Scap^I414C/L531C^. The control in *lane 1* with no Scap was transfected with 2 μg of pTK-Insig-2-6xMyc and 4 µg of pcDNA3 empty vector plasmid. The transfected cells were then depleted of cholesterol and replenished with the indicated concentrations of cholesterol (delivered in complexes with MCD), after which the cells were harvested and processed for coimmunoprecipitation assays and immunoblot analysis as described in *SI Appendix*, *Methods*. (*D*) Comparison of the ability of WT and mutant Scap (I414C/L531C) to mediate SREBP2 cleavage. Scap-deficient SRD-13A cells were set up and transfected as described in *SI Appendix*, *Methods* with 2 µg of pTK-3xFLAG-SREBP2, 1.5 µg of pTK-Insig-2-6xMyc, and 2 µg of either pTK-Scap^WT^ or pTK-Scap^I414C/L531C^. The transfected cells were then depleted of cholesterol and replenished with the indicated concentrations of cholesterol (delivered in complexes with MCD), after which cell lysates were analyzed by immunoblot as described in *SI Appendix*, *Methods*. *P*, precursor form of SREBP2; *N*, cleaved nuclear form of SREBP2.

**Fig. 7.**
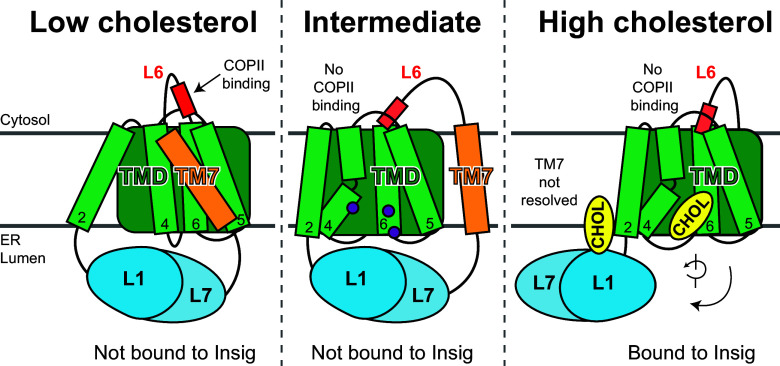
An updated model for cholesterol-mediated conformational changes in Scap. (*Left*) When ER cholesterol is low, the L1–L7 domain of Scap (*blue*) is oriented below its transmembrane domain (*green*) and TM7 (*orange*) is packed against a surface formed by TM4/TM5/TM6, resulting in exposure of a hexapeptide in L6 (red bar) for binding to the COPII machinery allowing for transport of Scap from ER to Golgi. (*Right*) When ER cholesterol rises above a threshold, the L1–L7 domain swings outward to be oriented below the ER membrane and TM7 moves away from TM4/TM5/TM6, allowing for Insig to bind to this surface. These events are accompanied by cholesterol binding to the L1–L7 domain as well as to the interface between TM4/TM5/TM6 and Insig, changing the disposition of the COPII-binding motif on L6, and thus halting Scap’s transport to Golgi. (*Middle*) In between these two extreme conformations is a hypothetical intermediate state where the L1–L7 domain remains under the transmembrane domain, TM7 is displaced from TM4/TM5/TM6, and yet the COPII-binding motif on L6 is inaccessible, preventing Scap transport to Golgi. Four point mutations to three residues (*purple circles*) cause Scap to adopt this intermediate state. This updated model highlights the modular and dynamic nature of Scap in membranes that allows it to rapidly respond to changes in cholesterol levels.

While the above analysis shows the effects of dissociating TM7 from the TM4/TM5/TM6 surface of Scap, we also devised strategies to lock TM7 against the same surface. If this strategy was successful, we would observe transport of Scap and subsequent SREBP cleavage even in the presence of added cholesterol. We considered the possibility of introducing cysteines at this binding interface to potentially engineer disulfide bonds (31) which would lock TM7 in place. After close inspection of the AlphaFold2 model of hamster Scap, we cast a wide net and chose nine residues on Scap’s TM7 and seven residues on Scap’s TM4/TM5/TM6 surface (Fig. 6*A*). We generated mutant Scaps where one cysteine was introduced in TM7 and another cysteine was introduced at the TM4/TM5/TM6 interface (*dashed lines*, Fig. 6*A*). We then transfected these mutant Scaps into Scap-deficient cells, along with Insig-2 and SREBP2. After transfection, the cells were treated with a high concentration of lipoproteins to increase their cholesterol levels, after which we assessed SREBP2 cleavage. As expected, minimal cleavage was observed when the cells contained WT Scap (*lane 1*, Fig. 6*B*). Of all the mutant Scaps with cysteine pairs, one pair at the ER luminal interface (oxidizing environment), I414C and L531C, resulted in SREBP2 cleavage even in the presence of cholesterol (*lane 12*, Fig. 6*B*). The failure of the other double cysteine mutants to promote SREBP2 cleavage in the presence of cholesterol (*lanes 2*–*11*, Fig. 6*B*) could be related to the lack of proximity and/or ideal geometry of these cysteine residues to promote a disulfide bond or more likely the challenge of forming disulfides in the membrane core due to lack of oxidizing equivalents (32). We next studied the properties of the I414C/L531C mutant of Scap in more detail. In contrast to WT Scap, the I414C/L531C mutant version of Scap did not interact with Insig-2, even at the highest concentrations of added cholesterol (*compare lanes 7*–*11 to 2*–*6*, Fig. 6*C*). Consistent with its inability to bind Insigs, the I414C/L531C mutant Scap failed to respond to cholesterol and continued to transport to Golgi as evidenced by uninhibited cleavage of SREBP2 (*compare lanes 6*–*10 to lanes 1*–*5*, Fig. 6*D*). To investigate whether the failure of I414C/L531C mutant Scap to respond to cholesterol was due to the formation of a disulfide bond, we made individual mutations where I414 and L531 were each mutated to either cysteine or alanine. None of these single mutants or the double alanine mutant had the same functional effect as the double cysteine mutant (*SI Appendix*, Fig. S10), further supporting our interpretation that a disulfide linkage prevents displacement of TM7. Combined with the results of Fig. 5 *E* and *F*, these studies support a model (Fig. 7) where TM7’s packing against the TM4/TM5/TM6 surface of Scap is in competition with the cholesterol-mediated association of Insig with Scap.

## Discussion

Understanding how cholesterol binding to Scap is transmitted across the lipid bilayer to disrupt the interaction between Scap’s cytosolic loop 6 (L6) and cytosolic COPII proteins has been a confounding problem (3). The current study provides a potential solution. Transmission of the cholesterol signal across the ER membrane is orchestrated by Scap’s TM7, which directly connects Scap’s luminal L1–L7 domain to its cytosolic L6. When ER cholesterol is low, the L1–L7 domain is positioned under the transmembrane domain (TMD), TM7 is tightly packed against a surface of the TMD composed of TM4/TM5/TM6, and L6 adopts a conformation where it can bind COPII proteins (*Left panel*, Fig. 7). When ER cholesterol rises above a threshold concentration of ~5 mol% of total ER lipids (4), the disposition of these three elements of Scap is dramatically altered. The L1–L7 domain rotates by 215° such that it is positioned under the membrane and not under the TMD, TM7 is no longer tightly packed against the TMD, and L6’s conformation changes to prevent its binding to COPII proteins (*Right panel*, Fig. 7). In this high-cholesterol state, the TM7-binding site on the TMD is instead occupied by Insig and a cholesterol molecule (Fig. 2*D*). Thus, Insig and cholesterol are in competition with TM7 for binding the same surface on Scap’s TMD (*Right panel*, Fig. 7).

While our structures reveal the two conformational extremes of Scap (low-cholesterol and high-cholesterol), insights into how changes in ER cholesterol trigger the conversion of Scap from one conformation to the other emerge from our extensive mutagenesis studies (Figs. 3–6). In our model, Scap samples the low-cholesterol and high-cholesterol conformations we have elucidated (Fig. 7). When in the high-cholesterol conformation, if the extended L1–L7 domain encounters accessible cholesterol on the ER luminal membrane surface (26), or if the TMD surface (made bare by movement of TM7) encounters ER bilayer cholesterol, then this conformation is stabilized. Further stabilization of this conformation of Scap is imparted by the binding of Insigs to the TMD surface. The interfacial sterol-binding site in the membrane between Scap and Insig is reminiscent of a molecular glue (33) that can further enhance the affinity of Scap for cholesterol and Insig. The net result of the cholesterol-triggered changes in Scap is to alter its L6 conformation, which precludes binding of COPII proteins (11), thus blocking transport of Scap from ER to Golgi. In contrast, if the L1–L7 domain or the TMD surface do not encounter cholesterol, Scap returns to its low-cholesterol conformation where TM7 snugly packs against the TMD, placing L6 in a conformation permissive to bind COPII proteins, thereby promoting Scap’s transport from ER to Golgi. The mutational analysis of TM7 (Figs. 5 and 6) is consistent with this model. Mutations in TM7 that disrupt its interaction with the TMD lock Scap in its high-cholesterol conformation, even when cells are depleted of cholesterol (Fig. 5), and mutations that stabilize the TM7/TMD interaction lock Scap into its low-cholesterol conformation, even when cells are replete with cholesterol (Fig. 6). Mutational analysis of the TMD surface of Scap (Fig. 4) and the TMD-binding surface of Insig (*SI Appendix*, Fig. S8) also identify several mutants that lock this sensing machinery in either the low-cholesterol or high-cholesterol states (summarized in *SI Appendix*, Tables S2 and S3).

An interesting exception to the above analysis is provided by four mutations of Scap’s TMD surface—Y351A, I414W, F417A, and F417W—that lock Scap into a state intermediate between the low-cholesterol and high-cholesterol conformations (Fig. 4). These mutant Scaps fail to bind Insigs, a feature of the low-cholesterol conformation where TM7 is locked in place against the TMD; yet, these mutants do not transport from ER to Golgi, akin to the high-cholesterol conformation where L6 is in an orientation that precludes binding to COPII proteins. A speculative model of this form of Scap, intermediate between the low-cholesterol and high-cholesterol conformations, is shown in Fig. 7 (*Middle panel*). We are currently pursuing structures of these Scap mutants to further define the conformational substates of the dynamic Scap sensor.

The identification of a bound cholesterol at the Scap/Insig interface (sterol A, Fig. 2) expands our understanding of Scap’s cholesterol-sensing mechanisms. Previous studies have biochemically characterized a cholesterol-binding site in the L1–L7 domain that has an all-or-none requirement for the sterol 3-hydroxyl group to be in the β-stereo-orientation and the sterol iso-octyl sidechain to be unadorned by polar hydroxyl groups found in oxysterols such as 25HC (6, 13). However, the structural studies to date have not identified cholesterol bound to this domain (14, 24, 25). In contrast, cholesterol and related molecules containing a steroid nucleus, including 25HC, have been observed at the sterol A site in multiple structures. Our current structure reveals a strong density for the tetracyclic steroid nucleus of cholesterol that could be stabilized through interactions with I348, Y351, and F417 (Fig. 3*A*). We do not observe strong density for either the 3-hydroxyl group or the iso-octyl side chain. Modeling of these cholesterol moieties in accordance with the known orientation of cholesterol in membranes suggests that the 3-hydroxyl may be stabilized by favorable contacts with the polar residue E347 and the iso-octyl side chain may be buried among hydrophobic sidechains (Fig. 4*A*). Further work is needed to define the binding mechanisms of cholesterol to Scap’s L1–L7 domain, 25HC to Insig, and the interplay between these sterol-binding sites and the sterol A site. This will likely require structures in ER-mimicking lipid bilayers or in an intact cellular environment by cryoelectron tomography.

## Materials and Methods

Scap/Insig-2 complex was purified in GDN detergent containing saturating amounts of cholesterol and incubated with 4G10^Fab^, after which cryo-EM structure determination was carried out as described in *SI Appendix*. To assess the effects of Scap and Insig-2 mutations on SREBP2 activation, Scap-deficient and Insig-deficient cells were transfected with the indicated mutant versions of Scap or Insig-2, respectively, and after cholesterol depletion followed by replenishment, SREBP2 cleavage was assessed as described in *SI Appendix*. To assess the effects of mutations on cholesterol-mediated Scap/Insig-2 interaction, Scap-deficient or Insig-deficient cells were transfected with the indicated mutant versions of Scap or Insig-2, respectively, and after cholesterol depletion followed by replenishment, Scap/Insig-2 complex formation was assayed by coimmunoprecipitation as described in *SI Appendix*. All other information including a list of reagents used in this study and details of assays for ^3^H-cholesterol and Fab binding to Scap can be found in *SI Appendix*.

## Supplementary Material

Appendix 01 (PDF)

Dataset S01 (PDF)

Dataset S02 (PDF)

Dataset S03 (PDF)

## Data Availability

Structural data have been deposited in the Protein Data Bank (PDB) and the Electron Microscopy Data Bank (EMDB). Structural models have been deposited in the Protein Data Bank (PDB) with coordinate accession numbers 9PXB (34), 9PY6 (35), and 9PY7 (36). Cryo-EM maps have been deposited in the Electron Microscopy Data Bank (EMDB) with accession numbers EMD-71964 (37), EMD-72010 (38), EMD-72012 (39), and EMD-72029 (40). All other study data are included in the manuscript and/or supporting information.

## References

[1] M. S. Brown, J. L. Goldstein, The SREBP pathway: Regulation of cholesterol metabolism by proteolysis of a membrane-bound transcription factor. Cell **89**, 331–340 (1997). 9150132 10.1016/s0092-8674(00)80213-5

[2] J. D. Horton, J. L. Goldstein, M. S. Brown, SREBPs: Activators of the complete program of cholesterol and fatty acid synthesis in the liver. J. Clin. Invest. **109**, 1125–1131 (2002). 11994399 10.1172/JCI15593 PMC150968

[3] M. S. Brown, A. Radhakrishnan, J. L. Goldstein, Retrospective on cholesterol homeostasis: The central role of Scap. Annu. Rev. Biochem. **87**, 783–807 (2018). 28841344 10.1146/annurev-biochem-062917-011852 PMC5828883

[4] A. Radhakrishnan, J. L. Goldstein, J. G. McDonald, M. S. Brown, Switch-like control of SREBP-2 transport triggered by small changes in ER cholesterol: A delicate balance. Cell Metab. **8**, 512–521 (2008). 19041766 10.1016/j.cmet.2008.10.008 PMC2652870

[5] J. D. Horton , Combined analysis of oligonucleotide microarray data from transgenic and knockout mice identifies direct SREBP target genes. Proc. Natl. Acad. Sci. U.S.A. **100**, 12027–12032 (2003). 14512514 10.1073/pnas.1534923100 PMC218707

[6] A. Radhakrishnan, L.-P. Sun, H. J. Kwon, M. S. Brown, J. L. Goldstein, Direct binding of cholesterol to the purified membrane region of SCAP: Mechanism for a sterol-sensing domain. Mol. Cell **15**, 259–268 (2004). 15260976 10.1016/j.molcel.2004.06.019

[7] M. Motamed , Identification of luminal loop 1 of Scap as the sterol sensor that maintains cholesterol homeostasis. J. Biol. Chem. **286**, 18002–18012 (2011), 10.1074/jbc.m111.238311. 21454655 PMC3093874

[8] Y. Zhang , Direct demonstration that Loop1 of Scap binds to Loop7 a crucial event in cholesterol homeostasis*. J. Biol. Chem. **291**, 12888–12896 (2016). 27068746 10.1074/jbc.M116.729798 PMC4933461

[9] T. Yang , Crucial step in cholesterol homeostasis: Sterols promote binding of SCAP to INSIG-1, a membrane protein that facilitates retention of SREBPs in ER. Cell **110**, 489–500 (2002). 12202038 10.1016/s0092-8674(02)00872-3

[10] L.-P. Sun, L. Li, J. L. Goldstein, M. S. Brown, Insig required for sterol-mediated inhibition of Scap/SREBP binding to COPII proteins in vitro. J. Biol. Chem. **280**, 26483–26490 (2005). 15899885 10.1074/jbc.M504041200

[11] L.-P. Sun, J. Seemann, J. L. Goldstein, M. S. Brown, Sterol-regulated transport of SREBPs from endoplasmic reticulum to Golgi: Insig renders sorting signal in Scap inaccessible to COPII proteins. Proc. Natl. Acad. Sci. U.S.A. **104**, 6519–6526 (2007). 17428919 10.1073/pnas.0700907104 PMC1851663

[12] C. M. Adams , Cholesterol and 25-hydroxycholesterol inhibit activation of SREBPs by different mechanisms, both involving SCAP and Insigs. J. Biol. Chem. **279**, 52772–52780 (2004). 15452130 10.1074/jbc.M410302200

[13] A. Radhakrishnan, Y. Ikeda, H. J. Kwon, M. S. Brown, J. L. Goldstein, Sterol-regulated transport of SREBPs from endoplasmic reticulum to Golgi: Oxysterols block transport by binding to Insig. Proc. Natl. Acad. Sci. U.S.A. **104**, 6511–6518 (2007). 17428920 10.1073/pnas.0700899104 PMC1851665

[14] D. L. Kober , Scap structures highlight key role for rotation of intertwined luminal loops in cholesterol sensing. Cell **184**, 3689–3701.e22 (2021). 34139175 10.1016/j.cell.2021.05.019 PMC8277531

[15] X. Wu, R. Yan, P. Cao, H. Qian, N. Yan, Structural advances in sterol-sensing domain-containing proteins. Trends Biochem. Sci. **47**, 289–300 (2022). 35012873 10.1016/j.tibs.2021.12.005

[16] H. Chen , Regulated degradation of HMG CoA reductase requires conformational changes in sterol-sensing domain. Nat. Commun. **13**, 4273 (2022). 35879350 10.1038/s41467-022-32025-5 PMC9314443

[17] X. Li , Structure of human Niemann-Pick C1 protein. Proc. Natl. Acad. Sci. U.S.A. **113**, 8212–8217 (2016). 27307437 10.1073/pnas.1607795113 PMC4961162

[18] C.-S. Huang , Cryo-EM structures of NPC1L1 reveal mechanisms of cholesterol transport and ezetimibe inhibition. Sci. Adv. **6**, eabb1989 (2020). 32596471 10.1126/sciadv.abb1989 PMC7304964

[19] X. Qi, P. Schmiege, E. Coutavas, J. Wang, X. Li, Structures of human Patched and its complex with native palmitoylated sonic hedgehog. Nature **560**, 128–132 (2018). 29995851 10.1038/s41586-018-0308-7 PMC6341490

[20] C. Siebold, R. Rohatgi, The inseparable relationship between cholesterol and hedgehog signaling. Annu. Rev. Biochem. **92**, 273–298 (2023). 37001135 10.1146/annurev-biochem-052521-040313 PMC10330520

[21] Y. Zhang, M. Motamed, J. Seemann, M. S. Brown, J. L. Goldstein, Point mutation in luminal loop 7 of Scap protein blocks interaction with loop 1 and abolishes movement to Golgi*. J. Biol. Chem. **288**, 14059–14067 (2013). 23564452 10.1074/jbc.M113.469528 PMC3656263

[22] R. B. Rawson, R. DeBose-Boyd, J. L. Goldstein, M. S. Brown, Failure to cleave sterol regulatory element-binding proteins (SREBPs) causes cholesterol auxotrophy in Chinese hamster ovary cells with genetic absence of SREBP cleavage-activating protein. J. Biol. Chem. **274**, 28549–28556 (1999). 10497220 10.1074/jbc.274.40.28549

[23] J. D. Feramisco , Intramembrane aspartic acid in SCAP protein governs cholesterol-induced conformational change. Proc. Natl. Acad. Sci. U.S.A. **102**, 3242–3247 (2005). 15728349 10.1073/pnas.0500206102 PMC552931

[24] R. Yan , A structure of human Scap bound to Insig-2 suggests how their interaction is regulated by sterols. Science **263**, eabb2224 (2021). 10.1126/science.abb2224 33446483

[25] R. Yan , Structural basis for sterol sensing by Scap and Insig. Cell Rep. **35**, 109299 (2021). 34192549 10.1016/j.celrep.2021.109299

[26] S. Xu , A cholesterol-binding bacterial toxin provides a strategy for identifying a specific Scap inhibitor that blocks lipid synthesis in animal cells. Proc. Natl. Acad. Sci. U.S.A. **121**, e2318024121 (2024). 38330014 10.1073/pnas.2318024121 PMC10873635

[27] A. Nohturfft, M. S. Brown, J. L. Goldstein, Sterols regulate processing of carbohydrate chains of wild-type SREBP cleavage-activating protein (SCAP), but not sterol-resistant mutants Y298C or D443N. Proc. Natl. Acad. Sci. U.S.A. **95**, 12848–12853 (1998). 9789003 10.1073/pnas.95.22.12848 PMC23627

[28] J. D. Feramisco, J. L. Goldstein, M. S. Brown, Membrane topology of human Insig-1, a protein regulator of lipid synthesis. J. Biol. Chem. **279**, 8487–8496 (2004). 14660594 10.1074/jbc.M312623200

[29] P. C. W. Lee, N. Sever, R. A. DeBose-Boyd, Isolation of sterol-resistant Chinese hamster ovary cells with genetic deficiencies in both Insig-1 and Insig-2. J. Biol. Chem. **280**, 25242–25249 (2005). 15866869 10.1074/jbc.M502989200

[30] J. Jumper , Highly accurate protein structure prediction with AlphaFold. Nature **596**, 583–589 (2021). 34265844 10.1038/s41586-021-03819-2 PMC8371605

[31] A. A. Dombkowski, K. Z. Sultana, D. B. Craig, Protein disulfide engineering. FEBS Lett. **588**, 206–212 (2014). 24291258 10.1016/j.febslet.2013.11.024

[32] L. Wang, C. Wang, Oxidative protein folding fidelity and redoxtasis in the endoplasmic reticulum. Trends Biochem. Sci. **48**, 40–52 (2023). 35871147 10.1016/j.tibs.2022.06.011

[33] S. Cao , Defining molecular glues with a dual-nanobody cannabidiol sensor. Nat. Commun. **13**, 815 (2022). 35145136 10.1038/s41467-022-28507-1 PMC8831599

[34] B. Williams , Hamster Scap/Insig-2 complex with cholesterol and bound Fab4G10. Protein Data Bank. http://www.rcsb.org/structure/9PXB. Deposited 5 August 2025.

[35] B. Williams , Hamster Scap/Insig-2 complex L1-L7 domain/Fab4G10 focused map. Protein Data Bank. http://www.rcsb.org/structure/9PY6. Deposited 7 August 2025.

[36] B. Williams , Hamster Scap L1-L7 domain/Fab4G10 focused map. Protein Data Bank. http://www.rcsb.org/structure/9PY7. Deposited 7 August 2025.

[37] B. Williams , Hamster Scap/Insig-2 complex with cholesterol and bound Fab4G10. Electron Microscopy Data Bank. http://www.ebi.ac.uk/emdb/EMD-71964. Deposited 5 August 2025.

[38] B. Williams , Hamster Scap/Insig-2 complex L1-L7 domain/Fab4G10 focused map. Electron Microscopy Data Bank. http://www.ebi.ac.uk/emdb/EMD-72010. Deposited 7 August 2025.

[39] B. Williams , Hamster Scap L1-L7 domain/Fab4G10 focused map. Electron Microscopy Data Bank. http://www.ebi.ac.uk/emdb/EMD-72012. Deposited 7 August 2025.

[40] B. Williams , Hamster Scap with bound Fab4G10. Electron Microscopy Data Bank. http://www.ebi.ac.uk/emdb/EMD-72029. Deposited 7 August 2025.

