# Supplementary material for: Competition between a transmembrane helix on Scap and a membrane cholesterol regulates Scap–Insig interaction and SREBP activation
Source: Proc Natl Acad Sci U S A. 2025 Dec 31;123(1):e2525043123. doi: 10.1073/pnas.2525043123 (PMC12773782; doi:10.1073/pnas.2525043123)
Supplement: Supplementary file 1 — Appendix 01 (PDF) [file pnas.2525043123.sapp.pdf]

PNAS

Supporting Information for

Competition between a transmembrane helix on Scap and a membrane cholesterol regulates Scap-Insig interaction and SREBP activation

Blake C. Williams[a], Daniel L. Kober[b], Xiao-chen Bai[a], Daniel M. Rosenbaum[a]*, and Arun Radhakrishnan[c]*

Departments of Biophysics[a], Biochemistry[b], and Molecular Genetics[c], University of Texas Southwestern Medical Center, Dallas, Texas 75390

* For correspondence:

dan.rosenbaum@utsouthwestern.edu or arun.radhakrishnan@utsouthwestern.edu

This PDF file includes:

Methods
List of key reagents
Figures S1 to S10
Tables S1 to S3
SI References

Methods

Cell Lines

HEK293S GnTI$^-$ cells were obtained from ATCC (CRL-3022) and maintained in suspension culture (130 rpm) in medium G at 37°C in 5% CO2 and 70% humidity, as described previously(1). Scap-deficient CHO cells (SRD-13A) were maintained in monolayer culture in medium B at 37°C at 8-9% CO2, as described previously(2). Insig-1/Insig-2-deficient CHO cells (SRD-15) were maintained in monolayer culture in medium F at 37°C in 8-9% CO2, as described previously(3). *Spodoptera frugiperda* (Sf9) cells were maintained in suspension culture (120 rpm) in SF900 III SFM media (Gibco) at 27°C. To guard against potential genomic instability, all cell lines were passaged for less than 6 weeks, after which a fresh aliquot of cells was thawed and propagated. All cell lines were confirmed to be free of mycoplasma contamination using the LookOut® Mycoplasma PCR Detection Kit (Sigma).

Reagents

Buffers and Solutions

IP buffer contains 50 mM HEPES-NaOH pH 7.5, 100 mM NaCl, 0.5% (w/v) CHAPS, 1 mM EDTA, and protease inhibitors [4 µg/mL aprotinin, 0.4 µg/mL leupeptin, 2 µg/mL pepstatin A, 0.2 mM PMSF, and 3 µM E64]. SDS Buffer contains 10 mM Tris-HCl pH 6.8, 100 mM NaCl, 1% (w/v) SDS, 1 mM EDTA, 1 mM EGTA, and protease inhibitors [13 µg/mL aprotinin, 1 µg/mL leupeptin, 5 µg/mL pepstatin A, 0.7 mM PMSF, and 10 µM E64]. SDS loading buffer contains 250 mM Tris-HCl (pH 6.8), 10% (w/v) SDS, 0.2% (w/v) bromophenol blue, 25% (v/v) glycerol, and 5% (v/v) β-mercaptoethanol.

Culture Media

Medium A is a 1:1 mixture of DMEM and Ham's F-12 medium supplemented with 5% (v/v) FCS, 100 units/mL penicillin, and 100 µg/mL streptomycin sulfate. Medium B is medium A supplemented with 1 mM sodium mevalonate, 20 µM sodium oleate, and 5 µg/mL cholesterol. Medium C is a 1:1 mixture of DMEM and Ham's F-12 medium supplemented with 5% (v/v) LPDS, 100 units/mL penicillin, and 100 µg/mL streptomycin sulfate. Medium D is medium C supplemented with 10 µM sodium compactin and 50 µM sodium mevalonate. Medium E is medium C supplemented with 50 µM sodium compactin and 50 µM sodium mevalonate. Medium F is medium C supplemented with 10 µM SR-12813 and 1 µg/mL 25-hydroxycholesterol. Medium G is FreeStyle 293 Medium (Thermo Fisher) supplemented with 2% v/v FCS, 100 units/mL penicillin, and 100 µg/mL streptomycin sulfate.

Plasmids

Constructs for mammalian cell protein expression and purification

pEZT-4G10^{Fab}-His$_{10}$ encodes, in order from its NH_2-terminus, the mouse IgK leader sequence (METDTLLLWVLLLWVPGSTGD), followed by 4G10^{Fab} Heavy chain sequence (TGVHSEVQLQQSGAELVRPGASVKLSCTASGFKIKDDYIHWVKQRPEQGLEWIGRIDP ANGHTRYAPKFQDKATITADTSSNTAYLQLSSLTSEDTAVYYCTRYNDYDAFYFDYWG

QGTTLTVSSASTKGPSVFPLAPSSKSTSGGTAALGCLVKDYFPEPVTVSWNSGALTSG VHTFPAVLQSSGLYSLSSVVTVPSSSLGTQTYICNVNHKPSNTKVDKRVEPKSCDKTGS G), a 22-aa linker sequence (GSGATNFSLLKQAGDVEENPG^P) containing the 2A "cleavage" peptide(4) from Porcine teschovirus (P2A), another mouse IgK leader sequence (METDTLLLWVLLLWVPGSTGD), 4G10^{Fab} Light chain sequence (TGVHSDIQMTQTTSSLSASLGDRVTISCRASQDIRNYLNWYQQKPDGTVKLLIYYTSRL HSGVPSRFSGSGSGTDYSLTISNLEQEDIATYFCQQTNTLPWTFGGGTKLEIKRTVAAP SVFIFPPSDEQLKSGTASVVCLLNNFYPREAKVQWKVDNALQSGNSQESVTEQDSKDS TYSLSSTLTLSKADYEKHKVYACEVTHQGLSSPVTKSFNRGEC), followed by a His_{10} tag. The ^ denotes the break in the peptide chain caused by the 2A sequence leading to the production of two separate peptides. The codon-optimized sequence of this construct was produced as a dsDNA fragment and assembled into a NheI and KpnI-linearized pEZT vector.

pEZT-His_{10}-$Scap^{WT}$(L1-L7) encodes, in order from its NH_2-terminus, the 21-aa honeybee melittin secretion signal (MKFLVNVALVFMVVYISYIYA), followed by a 4-aa linker (DYSS), a His_{10} tag, a 10-aa linker (DYDIPTTAMG), hamster Scap (P97260.1) Loop 1 residues 46-284, a 15-aa linker sequence that includes a TEV protease cleavage site (SENLYFQGSSGSSGS), and hamster Scap (P97260.1) Loop 7 residues 535-710. This construct is expressed downstream of the strong cytomegalovirus (CMV) promoter. The coding sequence was amplified from pBac-His_{10}-Scap(L1-L7)(5) and assembled into a NheI- and KpnI-linearized pEZT vector(6) using NEBuilder HiFi DNA assembly kit according to the manufacturer's instructions.

pEZT-His_{10}-$Scap^{EM}$(L1-L7) is a modified version of pEZT-His_{10}-$Scap^{WT}$(L1-L7) containing 7 mutations (V133A, T140V, H244D, A245S, K246R, A253S and M256K). These point mutations were introduced into the pEZT-His_{10}-$Scap^{WT}$(L1-L7) fusion construct using site-directed mutagenesis.

pEZT-His_8-$Scap^{WT}$-3xFLAG encodes, in order from its NH_2-terminus, a His_8 tag, hamster Scap (P97260.1, residues 2-1276) followed by 3 copies of the FLAG epitope DYKDDDDKGSDYKDDDDKGSDYKDDDDK. This construct is expressed downstream of the strong CMV promoter. The Scap coding sequence was amplified from pTK-Scap(7) and assembled into a XhoI- and KpnI-linearized pEZT vector using NEBuilder HiFi DNA assembly kit according to the manufacturer's instructions.

pEZT-His_8-$Scap^{EM}$-3xFLAG is a modified version of pEZT-His_8-$Scap^{WT}$-3xFLAG containing 7 mutations (V133A, T140V, H244D, A245S, K246R, A253S and M256K). These point mutations were introduced into the pEZT-His_8-$Scap^{WT}$-3xFLAG construct using site-directed mutagenesis. pEZT-His_8-$Scap^{I348A/Y351A/F417A}$-3xFLAG and pEZT-His_8-$Scap^{I348W/Y351W/F417W}$-3xFLAG are modified versions of pEZT-His_8-$Scap^{WT}$-3xFLAG containing mutations (I348A, Y351A, and F417A) or (I348W, Y351W, and F417W). These point mutations were introduced into the pEZT-His_8-$Scap^{WT}$-3xFLAG construct using site-directed mutagenesis.

pEZT-3xFLAG-TEV-His_8-$Scap^{EM}$(TM1-8) encodes in order from its NH_2-terminus, 3 copies of the FLAG epitope MDYKDDDDKGSDYKDDDDKGSDYKDDDDK, a 9-aa linker that

includes a TEV protease cleavage site (ENLYFQGTG), a His$_8$ tag, and Scap^{EM} residues 2-752. This construct was generated by amplifying the His$_8$ tag and Scap residues 2-752 from pEZT-His$_8$-Scap^{EM}-3xFLAG and inserting them into a pEZT vector that had been modified to include an N-terminal 3xFLAG-TEV motif followed by a TG spacer.

pEZT-Insig-2 encodes hamster Insig-2 (AAN28330.1, residues 1-225). The codon-optimized sequence of this construct was produced as a synthetic dsDNA fragment (gBlocks, IDT) and assembled into a NheI- and NotI-linearized pEZT vector.

Constructs for SREBP2 cleavage and Scap-Insig co-immunoprecipitation assays

pTK-3xFLAG-SREBP2 is an expression plasmid under control of the thymidine kinase (TK) promoter that encodes, in sequential order from the NH2-terminus, a 3xFLAG epitope tag (MDYKDDDDKGDYKDDDDKGDYKDDDDK), a 6-aa linker (IDGTVP), and human SREBP2 (aa 14-1141). An expression vector encoding HSV-tagged human SREBP2 (aa 14-1141)(8) served as a template and standard cloning methods were used to replace the HSV-epitope tag with a 3xFLAG epitope tag.

pTK-Insig-2-6xMyc has been described previously(9), it is an expression plasmid under control of the TK promoter that encodes human Insig-2 (aa 1-225) followed by six tandem copies of a c-Myc epitope tag (EQKLISEEDL). Point mutations were introduced into this construct using site-directed mutagenesis. pTK-Scap has been described previously(7), it is an expression plasmid under control of the TK promoter that encodes hamster Scap (aa 1-1276). Point mutations were introduced into this construct using site-directed mutagenesis.

pTK-3xHA-Scap encodes in order from its NH$_2$-terminus, 3 copies of the HA epitope MYPYDVPDYAGSYPYDVPDYAGSYPYDVPDYAGS followed by hamster Scap (aa 2-1276). Three HA-tags were introduced onto pTK-Scap using site-directed mutagenesis.

Construct validation

All plasmid open reading frames were verified by Sanger and/or full vector sequencing.

Assays

SREBP2 cleavage

For a typical assay to assess the effects of Scap mutations, Scap-deficient SRD-13A cells were set up on day 0 in 10 mL of medium B at a density of 7 x 10^5 cells per 10-cm dish. On day 1, media was removed, cells were washed with 5 mL of PBS, and 10 mL of medium A was added to each dish. Cells were then transfected with the indicated DNA plasmids (see Figure Legends) complexed with X-tremeGENE HP transfection reagent using a 3:1 µL reagent:µg DNA ratio in Opti-MEM according to the manufacturer's instructions. On day 2, the transfection medium was removed, cells were washed with 5 mL of PBS, after which 6 mL of cholesterol-depleting medium E supplemented with 2-hydroxypropyl-β-cyclodextrin (HPCD, 2% w/v) was added to each dish. After incubation at 37°C for 1h, the media was removed, cells were washed with 5 mL of PBS, following which 6 mL of medium E containing varying concentrations of complexes of cholesterol with methyl-β-cyclodextrin (MCD)(10) was added to each dish. After incubation at 37°C for 4 h, cells were washed with 5 mL of PBS, after which 1 mL of SDS Buffer was added to each dish.

After vigorous shaking for 45 min, a fraction of each cell lysate (400 µL) was removed, supplemented with 100 µL of SDS Loading buffer, heated at 55°C for 20 min, and evaluated by immunoblot analysis as described below. In some experiments this typical assay was slightly modified. For **Fig. 5E**, after incubation at 37°C for 1h in 6 mL of cholesterol-depleting medium E supplemented with HPCD (2% w/v) as above, the cells were not replenished with cholesterol/MCD. They were washed with 5 mL PBS, after which 1 mL of SDS Buffer was added to each dish. The rest of the experiment was carried out as above. For **Fig. 6B**, on day 2, the transfection medium was removed, cells were washed with 5 mL of PBS, after which 6 mL of fresh FCS-rich medium A was added to each dish. After incubation at 37°C for 4 h, cells were washed with 5 mL of PBS, after which 1 mL of SDS Buffer was added to each dish. The rest of the experiment was carried out as above.

For a typical assay to assess the effects of Insig-2 mutations, Insig-1/Insig-2-deficient SRD-15 cells were set up on day 0 in 10 mL of medium F at a density of 7 x 10^5 cells per 10-cm dish. On day 1, media was removed, cells were washed with 5 mL of PBS, and 10 mL of medium C was added to each dish. Cells were then transfected with the indicated DNA plasmids (see Figure Legends) complexed with X-tremeGENE HP transfection reagent using a 3:1 µL reagent:µg DNA ratio in Opti-MEM according to the manufacturer's instructions. On day 2, the transfection medium was removed, cells were washed with 5 mL of PBS, after which 6 mL of cholesterol-depleting medium E supplemented with HPCD (2% w/v) was added to each dish. After incubation at 37°C for 1h, the media was removed, cells were washed with 5 mL of PBS, following which 6 mL of medium E containing varying concentrations of cholesterol/MCD complexes was added to each dish. After incubation at 37°C for 4 h, cells were washed with 5 mL of PBS, after which 1 mL of SDS Buffer was added to each dish. After vigorous shaking for 45 min, a fraction of each cell lysate (400 µL) was removed, supplemented with 100 µL of SDS Loading buffer, heated at 55°C for 20 min, and evaluated by immunoblot analysis as described below.

Scap-Insig co-immunoprecipitation

For a typical assay to assess the effect of Scap mutations on the interaction between Scap and Insig, Scap-deficient SRD-13A cells were set up on day 0 in 10 mL of medium B at a density of 7 x 10^5 cells per 10-cm dish (two dishes per condition). On day 1, media was removed, cells were washed with 5 mL of PBS, and 10 mL of medium A was added to each dish. Cells were then transfected with the indicated DNA plasmids (see Figure Legends) complexed with X-tremeGENE HP transfection reagent using a 3:1 µL reagent:µg DNA ratio in Opti-MEM according to the manufacturer's instructions. On day 2, the transfection medium was removed, cells were washed with 5 mL of PBS, after which 6 mL of cholesterol-depleting medium E supplemented with HPCD (1 % w/v) was added to each dish. After incubation at 37°C for 1 h, the media was removed, cells were washed with 5 mL of PBS, following which 6 mL of medium E containing varying concentrations of cholesterol/MCD complexes was added to each dish. After incubation at 37°C for 2 h, the medium was removed, and 5 mL ice-cold PBS was added to each dish. Cells were then scraped into 50 mL centrifuge tubes, and after centrifugation at 1,000 x g for 5 min at 4°C, the cell pellets were resuspended in 1 mL IP Buffer with varying concentrations of

cholesterol/MCD complexes and transferred to 1.5 mL tubes. The resuspended cells were disrupted by passage through a 22-gauge needle 10 times and placed on a rotator at 4°C for 1 h. The cell lysates were then subjected to centrifugation at 1 x 10^5 x g for 30 min at 4°C, after which a fraction of each supernatant (80 µL), designated as "**Input**", was supplemented with 20 µL of SDS Loading buffer, heated at 55°C for 20 min, and saved for immunoblot analysis. The remainder of the supernatant (~920 µL) was transferred to a 1.5 mL tube containing 50 µL A/G agarose beads (that had been pre-equilibrated in IP buffer), and placed on a rotator at 4°C. After 1 h, the tubes were subjected to centrifugation at 1,000 x g for 5 min, and the supernatant was carefully transferred to a fresh 1.5 mL tube containing 50 µL A/G agarose beads (that had been pre-equilibrated in IP buffer), and 20 µg monoclonal IgG-9E10 (anti-Myc). The mixtures were placed on a rotator overnight (12-16 h) at 4°C. After centrifugation at 1,000 x g for 5 min, the supernatant was carefully discarded and 1 mL of IP Buffer with varying concentrations of cholesterol/MCD complexes was added to each tube. After incubation on a rotator for 20 min at 4°C, the mixtures were subjected to centrifugation at 1,000 x g for 5 min, following which the supernatant was carefully removed. This washing step was repeated two additional times. After the final wash step, bound proteins were eluted from the beads by resuspending with 100 µL SDS Buffer supplemented with 1 mM TCEP at room temperature. After 10 min, the mixtures were subjected to centrifugation at 2,000 x g for 5 min. A fraction of each supernatant (80 µL) containing the eluted proteins designated as "**Pellet**", was removed and supplemented with 20 µL of SDS Loading buffer, heated at 55°C for 20 min, and saved for immunoblot analysis.

For a typical assay to assess the effect of Insig mutations on the interaction between Scap and Insig, Insig-1/Insig-2-deficient SRD-15 cells were set up on day 0 in 10 mL of medium F at a density of 7 x 10^5 cells per 10-cm dish (two dishes per condition). On day 1, media was removed, cells were washed with 5 mL of PBS, and 10 mL of cholesterol-depleting medium D was added to each dish. Cells were then transfected with the indicated DNA plasmids (see Figure Legends) complexed with X-tremeGENE HP transfection reagent using a 3:1 µL reagent:µg DNA ratio in Opti-MEM according to the manufacturer's instructions. On day 2, the transfection medium was removed, cells were washed with 5 mL of PBS, after which 6 mL of cholesterol-depleting medium E supplemented with HPCD (1 % w/v) was added to each dish. After incubation at 37°C for 1 h, the media was removed, cells were washed with 5 mL of PBS, following which 6 mL of medium E containing varying concentrations of cholesterol/MCD complexes was added to each dish. After incubation at 37°C for 2 h, the medium was removed, and 5 mL ice-cold PBS was added to each dish. Cells were then scraped into 50 mL centrifuge tubes, and after centrifugation at 1,000 x g for 5 min at 4°C, the cell pellets were resuspended in 1 mL IP Buffer with varying concentrations of cholesterol/MCD complexes and transferred to 1.5 mL tubes. The resuspended cells were disrupted by passage through a 22-gauge needle 10 times and placed on a rotator at 4°C for 1 h. The cell lysates were then subjected to centrifugation at 1 x 10^5 x g for 30 min at 4°C, after which a fraction of each supernatant (80 µL), designated as "**Input**", was supplemented with 20 µL of SDS Loading buffer, heated at 55°C for 20 min, and saved for immunoblot analysis. The remainder of the supernatant (~920 µL) was transferred to a 1.5 mL tube containing 50 µL A/G agarose

beads (that had been pre-equilibrated in IP buffer), and placed on a rotator at 4°C. After 1 h, the tubes were subjected to centrifugation at 1,000 x g for 5 min, and the supernatant was carefully transferred to a fresh 1.5 mL tube containing 50 µL A/G agarose beads (that had been pre-equilibrated in IP buffer), and 20 µg monoclonal IgG-9E10 (anti-Myc). The mixtures were placed on a rotator overnight (12-16 h) at 4°C. After centrifugation at 1,000 x g for 5 min, the supernatant was carefully discarded and 1 mL of IP Buffer with varying concentrations of cholesterol/MCD complexes was added to each tube. After incubation on a rotator for 20 min at 4°C, the mixtures were subjected to centrifugation at 1,000 x g for 5 min, following which the supernatant was carefully removed. This washing step was repeated two additional times. After the final wash step, bound proteins were eluted from the beads by resuspending with 100 µL SDS Buffer supplemented with 1 mM TCEP at room temperature. After 10 min, the mixtures were subjected to centrifugation at 2,000 x g for 5 min. A fraction of each supernatant (80 µL) containing the eluted proteins designated as "**Pellet**", was removed and supplemented with 20 µL of SDS Loading buffer, heated at 55°C for 20 min, and saved for immunoblot analysis.

Immunoblot analysis
All samples were subjected to SDS-PAGE (4-15% gradient in **Fig. S7A**, 10% in all other Figures), after which proteins were transferred to nitrocellulose membranes using Trans-Blot Turbo Transfer System (BioRad Laboratories). After incubation with blocking buffer (PBS-Tween20 supplemented with 5% (w/v) non-fat milk) for 1 h at room temperature, the membranes were incubated with primary antibodies overnight at 4°C. The following primary antibodies, diluted in blocking buffer, were used: IgG-22D5 (5 µg/mL) to detect endogenous and overexpressed SREBP2, IgG-2G10 (5 µg/mL) to detect endogenous and overexpressed Scap, IgG-9E10 anti-Myc (5 µg/mL) to detect overexpressed Insig-2, anti-Actin (1:1000) to detect Actin, anti-FLAG M2 (5 µg/mL) to detect overexpressed SREBP2, anti-HA (1:1000) to detect overexpressed Scap. Bound antibodies were detected by chemiluminescence (SuperSignal West Pico Chemiluminescent Substrate, Thermo Scientific) using a 1:5000 dilution of either donkey or goat anti-mouse IgG (Jackson ImmunoResearch) or goat anti-rabbit IgG (Jackson ImmunoResearch) conjugated to horseradish peroxidase. Membranes were exposed to Pheonix Blue X-Ray Film (Pheonix Research Products) at room temperature for 1-300 s for all cases. Equal aliquots of cell lysates or eluates were evaluated by immunoblot analysis.

Fab binding to Scap
Binding between $4G10^{Fab}$ and $Scap^{WT}$(L1-L7) or $Scap^{EM}$(L1-L7) was assessed as follows: 88 µg of purified $Scap^{WT}$(L1-L7) or $Scap^{EM}$(L1-L7) was incubated with or without 132 µg of $4G10^{Fab}$ for 1 h, and then subjected to analytical gel filtration at a flow rate of 0.7 mL/min over a Superdex™ 200 Increase 10/300 column (GE Healthcare) equilibrated in 50 mM Tris-HCl pH 7.5, 150 mM NaCl. The absorbance at 280 nm was continuously monitored and aliquots of fractions were subjected to SDS-PAGE and Coomassie staining as indicated in the Figure Legends.

^{3}H-Cholesterol binding to Scap
Binding of ^{3}H-cholesterol to purified His_8-$Scap^{WT}$-3xFLAG, His_8-$Scap^{I348A/Y351A/F417A}$-3xFLAG, and His_8-$Scap^{I348W/Y351W/F417W}$-3xFLAG was measured as previously

described(1). Each reaction, in a final volume of 100 µL of binding buffer (50 mM HEPES-NaOH pH 7.5, 150 mM NaCl, 0.01% w/v LMNG, 0.002% w/v CHS, 0.002% Cholate, 0.5 mM TCEP) contained 2 µg of each of the purified proteins (13.9 pmol) and varying concentrations of ^{3}H-cholesterol (delivered in 3 µL of ethanol). After incubation for 4 h at 4°C, each reaction was loaded onto a column packed with 0.3 mL of Ni^{2+}-NTA-agarose beads pre-equilibrated with 2 mL binding buffer. The columns were washed with 3 mL of binding buffer, after which bound His-tagged Scap proteins were eluted with 1 mL of the same buffer containing 250 mM imidazole. Aliquots of the eluate (0.5 mL) were assayed for radioactivity in a liquid scintillation counter (Beckman Coulter LS 6500).

Reproducibility of assays
All cellular assays were carried out at least 3 times, using different batches of cells set up on different days. All ^{3}H-cholesterol binding assays were carried out in triplicate.

Protein Expression and Purification

Production of baculoviruses for protein overexpression
BacMam baculoviruses for mammalian protein expression from pEZT constructs were produced using the Bac-to-Bac expression system, and the GFP-expressing viruses were titered by serial dilution in Sf9 cells as previously described(6). Viruses were concentrated by centrifugation at 66,700 x g for 1 hr at 4°C to achieve titers necessary to infect HEK GnTI$^-$ cells at a multiplicity of infection (MOI) of 1-3 viruses per cell while ensuring that the volume of virus added to the final culture did not exceed 10% of the total volume.

Purification of 4G10^{Fab}
Each 800 mL culture of HEK293S GnTI$^-$ cells (2.5-3 x 10^6 cells/mL) was transduced with baculovirus for pEZT-4G10^{Fab}-His$_{10}$ at MOI of 1-3. At the time of transduction, the cultures were supplemented with 2.5 mM sodium butyrate to increase expression. Transduced cells were cultured for 60-72 h with orbital shaking at 130 rpm in an incubator maintained at 30°C, 8% CO2, and 70% humidity. Media containing secreted proteins was harvested by centrifugation at 4,000 x g for 10 min at 4°C, filtered through a 0.22 µm Vacuum Bottle Top Filter (Millipore), and stored at -80°C until purification.

All purification steps were conducted on ice or at 4°C. Media containing secreted proteins was thawed and loaded onto a column packed with 10 mL Ni^{2+}-NTA-sepharose beads (pre-equilibrated with 3 column volumes (CV) of PBS) at a flow-rate of 3-5 mL/min. The resin was then washed with 10 CV of Ni-wash buffer (50 mM HEPES-NaOH pH 7.5, 150 mM NaCl, and 50 mM imidazole), after which, bound proteins were eluted with Ni-wash buffer supplemented with 250 mM imidazole. Eluted proteins were concentrated to 1 mL using a 30kDa MWCO Amicon centrifugal filter and subjected to gel filtration at a flow rate of 0.7 mL/min over a Superdex™ 200 Increase 10/300 column (GE Healthcare) equilibrated in gel filtration buffer (50 mM HEPES-NaOH pH 7.5, 150 mM NaCl). Protein-rich fractions were pooled and either stored at 4°C before use or divided into 250 µg aliquots, frozen in liquid nitrogen, and stored at -80°C. A typical purification from an 800 mL culture yields 2-3 mg of 4G10^{Fab}.

Purification of Scap^{WT}(L1-L7) and Scap^{EM}(L1-L7)

Each 800 mL culture of HEK293S GnTI^{-} cells (2.5-3 x 10^6 cells/mL) was transduced with baculovirus for either pEZT-His$_{10}$-Scap^{WT}(L1-L7) or pEZT-His$_{10}$-Scap^{EM}(L1-L7) at MOI of 1-3. At the time of transduction, the cultures were supplemented with 2.5 mM sodium butyrate to increase expression. Transduced cells were cultured for 60-72 h with orbital shaking at 130 rpm in an incubator maintained at 30°C, 8% CO2, and 70% humidity. Media containing secreted proteins was harvested by centrifugation at 4,000 x g for 10 min at 4°C, filtered through a 0.22 µm Vacuum Bottle Top Filter (Millipore), and stored at -80°C until purification.

All purification steps were conducted on ice or at 4°C. Media containing secreted proteins was thawed and loaded onto a column packed with 7.5 mL Ni^{2+}-NTA-agarose beads (pre-equilibrated with 3 CV of PBS) at a flow-rate of 3-5 mL/min. The resin was then washed with 15 CV of Ni-wash buffer (50 mM Tris-HCl pH 7.5, 150 mM NaCl, 0.5 mM TCEP, and 50 mM imidazole), after which, bound proteins were eluted with Ni-wash buffer supplemented with 400 mM imidazole. Eluted proteins were concentrated to 1 mL using a 30kDa MWCO Amicon centrifugal filter and subjected to gel filtration at a flow rate of 0.7 mL/min over a Superdex^{TM} 200 Increase 10/300 column (GE Healthcare) equilibrated in 50 mM Tris-HCl pH 7.5, 150 mM NaCl. Protein-rich fractions were pooled and stored at 4°C before use in 4G10^{Fab} binding experiments.

Purification of Scap^{EM} and Scap^{EM}(TM1-8)/Insig-2

Each 800 mL culture of HEK293S GnTI^{-} cells (2.5-3 x 10^6 cells/mL) was transduced with either a single baculovirus (pEZT-His$_8$-Scap^{WT}-3xFLAG or pEZT-His$_8$-Scap^{EM}-3xFLAG at MOI of 1-3) or two baculoviruses (pEZT-Insig-2 and pEZT-3xFLAG-TEV-His$_8$-Scap^{EM}(TM1-8) at a ratio of 3:1). At the time of transduction, the cultures were supplemented with 2.5 mM sodium butyrate to increase expression. Transduced cells were cultured for 60-72 h with orbital shaking at 130 rpm in an incubator maintained at 30°C, 8% CO2, and 70% humidity. Cells were harvested by centrifugation at 4,000 x g for 10 min at 4°C, washed with PBS, and stored at -80°C until purification.

All purification steps were conducted on ice or at 4°C. Cell pellets were thawed, loosened by Dounce homogenization, and lysed in hypotonic buffer (10 mM HEPES-NaOH pH 7.5, 2.5 mM EDTA, 0.5 mM TCEP, and protease inhibitors [160 µg/mL benzamidine, 1 mM PMSF, 1 mM E64, and 2.5 µg/mL leupeptin]) while stirring for 30 min. Membranes from the lysed cells were pelleted by centrifugation at 21,000 x g for 30 min. Membrane pellets were loosened by Dounce homogenization and incubated in solubilization buffer (50 mM HEPES-NaOH pH 7.5, 500 mM NaCl, 20% v/v glycerol, 1% w/v LMNG, 0.2% w/v CHS, 0.2% w/v sodium cholate, 0.5 mM TCEP, 1 mU/µL benzonase, and protease inhibitors). After stirring at 500 rpm for 1 h, insoluble material was pelleted by centrifugation for 1 h at 1 x 10^5 x g at 4°C. The supernatant containing solubilized proteins was supplemented with additional protease inhibitors and incubated with 10 mL anti-FLAG M2 affinity resin on a rotator. After 3 h, the resin was collected by centrifugation at 1,000 x g for 5 min at 4°C, transferred to an empty column, and washed with 10 CV M2 wash buffer (50 mM HEPES-NaOH pH 7.5, 150 mM NaCl, 10% v/v glycerol, and 0.5 mM TCEP) supplemented with either 0.1% w/v GDN for Scap^{EM} or 0.1% w/v GDN saturated with 0.01% w/v cholesterol

for $Scap^{EM}$(TM1-8)/Insig-2. Bound proteins were eluted with each respective M2 wash buffer supplemented with 400 µg/mL FLAG peptide (DYKDDDDA, Bio-matik). Eluted proteins were concentrated to 1 mL using a 100k MWCO Amicon centrifugal filter and subjected to gel filtration at a flow rate of 0.7 mL/min over a Superose™ 6 Increase 10/300 column (GE Healthcare) equilibrated in gel filtration 1 buffer (20 mM HEPES-NaOH pH 7.5, 150 mM NaCl, 0.5 mM TCEP) supplemented with either 0.02% w/v GDN for $Scap^{EM}$ or 0.02% w/v GDN saturated with 0.002% w/v cholesterol for $Scap^{EM}$(TM1-8)/Insig-2. Protein-rich fractions were immediately used for cryo-EM studies. A typical purification from 6-10 x 800 mL cultures yielded 1-3 mg of $Scap^{EM}$ or $Scap^{EM}$(TM1-8)/Insig-2 complex.

Generating complexes of $Scap^{EM}$/4G10^{Fab} and $Scap^{EM}$(TM1-8)/Insig-2/4G10^{Fab} for cryo-EM

Purified proteins from above were incubated for 30 min on ice with excess 4G10^{Fab} (1.5-fold for fresh Fabs or 3-fold for Fabs thawed from -80°C stocks). The mixtures were concentrated to 1 mL using a 100k MWCO Amicon centrifugal filter, and then subjected to gel filtration at a flow rate of 0.7 mL/min over a Superose™ 6 Increase 10/300 column equilibrated in gel filtration 2 buffer (20 mM HEPES-NaOH pH 7.5, 150 mM NaCl, 0.5 mM TCEP) supplemented with either 0.004% w/v GDN for $Scap^{EM}$ or 0.004% w/v GDN saturated with 0.0004% w/v cholesterol for $Scap^{EM}$(TM1-8)/Insig-2. Peak fractions were concentrated to ~5 mg/mL using a Sartorius 100 kDa centrifugal concentrator. The final sample was subjected to centrifugation at 21,130 x g for 10 min at 4°C to remove insoluble material and then the sample was vitrified as below.

Purification of $Scap^{WT}$, $Scap^{I348A/Y351A/F417A}$, and $Scap^{I348W/Y351W/F417W}$ for cholesterol binding assays

Each 800 mL culture of HEK293S GnTI$^-$ cells (2.5-3 x 10^6 cells/mL) was transduced with baculovirus for pEZT-His_{10}-$Scap^{WT}$-3xFLAG, pEZT-His_{10}-$Scap^{I348A/Y351A/F417A}$-3xFLAG, or pEZT-His_{10}-$Scap^{I348W/Y351W/F417W}$-3xFLAG at MOI of 1-3. At the time of transduction, the cultures were supplemented with 2.5 mM sodium butyrate to increase expression. Transduced cells were cultured for 60-72 h with orbital shaking at 130 rpm in an incubator maintained at 30°C, 8% CO2, and 70% humidity. Cells were harvested by centrifugation at 4,000 x g for 10 min at 4°C, washed with PBS, and stored at -80°C until purification.

All purification steps were conducted on ice or at 4°C. Cell pellets were thawed, loosened by Dounce homogenization, and lysed in hypotonic buffer (10 mM HEPES-NaOH pH 7.5, 2.5 mM EDTA, 0.5 mM TCEP, and protease inhibitors [160 µg/mL benzamidine, 1 mM PMSF, 1 mM E64, and 2.5 µg/mL leupeptin]) while stirring for 30 min. Membranes from the lysed cells were pelleted by centrifugation at 21,000 x g for 30 min. Membrane pellets were loosened by Dounce homogenization and incubated in solubilization buffer (50 mM HEPES-NaOH pH 7.5, 500 mM NaCl, 20% v/v glycerol, 1% w/v LMNG, 0.2% w/v CHS, 0.2% w/v sodium cholate, 0.5 mM TCEP, 1 mU/µL benzonase, and protease inhibitors). After stirring at 500 rpm for 1 h, insoluble material was pelleted by centrifugation for 1 h at 1 x 10^5 x g at 4°C. The supernatant containing solubilized proteins was supplemented with additional protease inhibitors and incubated with 10 mL anti-FLAG M2 affinity resin on a rotator. After 3 h, the resin was collected by centrifugation at 1,000 x g for 5 min at 4°C, transferred to an empty column, and washed with 10 CV M2 wash buffer (50 mM HEPES-

NaOH pH 7.5, 150 mM NaCl, 10% v/v glycerol, and 0.5 mM TCEP) supplemented with 0.05% w/v LMNG, 0.01% w/v CHS, and 0.01% w/v sodium cholate. Bound proteins were eluted with M2 wash buffer supplemented with 400 µg/mL FLAG peptide (DYKDDDDA, Bio-matik). Eluted proteins were concentrated to 1 mL using a 100k MWCO Amicon centrifugal filter and subjected to gel filtration at a flow rate of 0.7 mL/min over a Superose^{TM} 6 Increase 10/300 column (GE Healthcare) equilibrated in gel filtration 1 buffer (20 mM HEPES-NaOH pH 7.5, 150 mM NaCl, 0.5 mM TCEP) supplemented with 0.01% w/v LMNG, 0.002% w/v CHS, and 0.002% w/v sodium cholate. Protein-rich fractions were pooled and stored on ice for use in ^{3}H-cholesterol binding assays. A typical purification from 6-10 x 800 mL cultures yielded 1-3 mg of Scap.

Cryo-Electron Microscopy

Grid preparation

Quantifoil® Au 300 mesh R 1.2/1.3 grids were plasma cleaned with air at 30 mA for 80s using a Pelco easiglow machine. 3 µL of Scap^{EM}/4G10^{Fab} or Scap^{EM}(TM1-8)/Insig-2/4G10^{Fab} samples were applied to grids at 4°C in 100% humidity and blotted for 4.5 s followed by freezing in liquid ethane cooled by liquid nitrogen, using a Vitrobot Mark IV. Grids were stored in liquid nitrogen until imaging.

Data collection

Micrographs were acquired on a Titan Krios G2 microscope (Thermo Fisher) with a K3 Summit direct electron detector (Gatan) in the super-resolution CDS counting mode, operated at 300 kV using SerialEM software. The calibrated magnification was set to 46,296 and 9 centered images were collected for each stage movement using the beam-tilt imaging method. The slit width of the GIF-Quantum energy filter was set to 20 eV. Micrographs were dose-fractioned into 50 frames at the dose rate of 1.2 $e^{-}/Å^{2}$/frame.

Image processing

Movies of Scap^{EM}/4G10^{Fab} using CryoSPARC v3.5.2(11), were subjected to Patch Motion Correction, where they were Fourier cropped to a pixel size of 1.07 Å, followed by Patch CTF Estimation. Manual blob picking was used to generate the initial templates and 4.2M particles were picked and extracted from 3834 micrographs. Initial particles were extracted and binned to a pixel size of 2.14 Å. After 3 rounds of 2D classification, 1M particles were selected and used to generate 4 ab-initio models. These particles and initial models were subjected to Heterogenous Refinement yielding a single class showing good secondary structure features. Particles from this class were subjected to another round of Heterogenous Refinement along with all 4 maps from the previous Heterogenous Refinement, yielding a single class with most of the particles. These particles were re-extracted to the original pixel size of 1.07 Å and subjected to Non-Uniform Refinement to produce a map of ~3 Å resolution with clear structural features on the L1-L7 domain and 4G10^{Fab} but lacking secondary structural features in the TM region and C-terminal domain. To improve the resolution for the L1-L7 domain, we performed Local Refinement by using a soft mask around L1-L7 domain and part of 4G10^{Fab} and subtracting the inverse volume from all the particles. The final reconstruction of the L1-L7 domain yielded a map resolved at 2.8 Å resolution.

Movie frames of $Scap^{EM}$(TM1-8)/Insig-2/4G10^{Fab} were motion-corrected, dose-weighted using MotionCor2(12) , and binned two-fold, resulting in a pixel size of 1.08 Å. CTF correction was then performed using Gctf(13) . The rest of the image processing steps were carried out using RELION 3.1(14) . Particles were first roughly picked by using the Laplacian-of-Gaussian blob method, and then subjected to 2D classification. Class averages representing projections of $Scap^{EM}$(TM1-8)/Insig-2/4G10^{Fab} in different orientations were used as templates for reference-based particle picking. A total of 16,886,990 particles were then picked from 26,612 micrographs. Particles were extracted and binned three times (leading to 3.24 Å/pixel) and subjected to two rounds of 2D classification. Particles in good 2D classes were chosen (9.9M) for 3D classification into 8 classes using an initial model generated from a subset of the particles. Particles from one of the 3D classes (2.12M) showing good secondary structure features were selected and re-extracted into the original pixel size of 1.08 Å. Subsequently we performed finer 3D classification into 6 classes by using local search in combination with small angular sampling. One resulting class (370,255 particles) showed improved density for the TM region, and had better refinement statistics (resolution, alignment accuracy) than all other individual classes or combinations thereof. Therefore, this single class was selected for further processing. To improve the resolution for the L1-L7 domain, we performed focused 3D refinement, along with CTF refinement and particle polishing, by using a soft mask around L1-L7 domain and part of 4G10^{Fab}. The final reconstruction of the L1-L7 domain was resolved at 2.9 Å resolution. To improve the resolution for the TM region, we performed a focused 3D classification with density subtraction(15) . In this approach, the density corresponding to the L1-L7 domain and 4G10^{Fab} as well as the detergent micelle was subtracted from the original particles. The subsequent 3D classification on the modified particles was carried out by applying a mask around the TM region and having all the orientations fixed at the value determined in the 3D refinement. This round of 3D classification yielded 4 classes of particles, among which one major class (160,849 particles) showed good density for the TM region and a higher particle number. 3D auto-refinement with RELION 5 blush regularization(16) of this unsubtracted class yielded a map at 3.2 Å resolution after CTF refinement and particle polishing. For the Figures, reconstructions were sharpened by DeepEMhancer(17).

Model Building

Initial models for human Scap/Insig-2 (7ETW)(18) and chicken Scap L1-L7 domain/4G10^{Fab} (7LKH and 7LKF)(1) were obtained from previous studies. Model building was done using WinCoot version 0.9.8.7(19) and the raw maps. The model was built through iterations of real-space refinement in Phenix version 1.20.1-4487(20) with secondary structure restraints and rounds of refinement in ISOLDE 1.6(21). Model geometrics were assessed using the MolProbity(22) module in Phenix 1.20.1-4487, the MolProbity 4.5.2 server and the PDB Validation Server V5.33/0.58. Refinement statistics are provided in **Table S1**. Figures were generated using ChimeraX version 1.6.1(23).

Quantification and Statistical Analysis

Cryo-EM data collection, analysis, and refinement statistics are shown in **Table S1**. For ^{3}H-cholesterol binding studies (**Fig. S7B**), error bars indicate standard error of the mean of three assays.

List of Key Reagents

REAGENT or RESOURCE	SOURCE	IDENTIFIER
**Antibodies**		
HRP-conjugated Donkey anti-mouse IgG	Jackson ImmunoResearch	Cat# 115-035-062
HRP-conjugated Goat anti-mouse IgG	Jackson ImmunoResearch	Cat# 115-035-003
HRP-conjugated Goat anti-rabbit IgG	Jackson ImmunoResearch	Cat# 111-035-045
Mouse monoclonal Anti-FLAG M2 clone (Hybridoma cells)	DSHB Hybridoma Product	Cat# 12C6c
Mouse monoclonal Anti-Myc (Hybridoma cells)	ATCC	Cat# CCL-1729, IgG-9E10
Mouse monoclonal Anti-Scap	Kober et al.,2020(24)	IgG-2G10
Rabbit monoclonal Anti-Actin	Sigma-Aldrich	Cat# A2066
Rabbit monoclonal Anti-HA	Cell Signaling	Cat# 3724
Rabbit monoclonal Anti-SREBP2	McFarlane et al., 2015(25)	IgG-22D5
**Bacterial and virus strains**		
MAX Efficiency DH10Bac Competent cells	Thermo Fisher	Cat# 10361012
XL10-Gold Ultracompetent cells	Agilent	Cat# 200317
**Chemicals, peptides, and recombinant proteins**		
[^{3}H]Cholesterol	American Radiolabeled Chemicals	Cat# ART 2133
β-mercaptoethanol	Sigma-Aldrich	Cat# M6250
25-Hydroxycholesterol	Steraloids	Cat# C6510-000
2-Hydroxypropyl-β-cyclodextrin (HPCD)	Sigma-Aldrich	Cat# 389145
5-Cholesten-3β-OL Hemmisuccinate (CHS)	Steraloids	Cat# C6823-000
Anti-FLAG M2 affinity resin	Sigma-Aldrich	Cat# A2220
Aprotinin	SelleckChem	Cat# S7377
Benzamidine hydrochloride monohydrate	Goldbio	Cat# B-050-250
Benzonase	EMDMillipore	Cat# 70746-3
Bromophenol blue	Sigma-Aldrich	Cat# 114391
CHAPS	Anatrace	Cat# C316
Cholesterol	Sigma	Cat# C8667
Dulbecco's Modified Eagle Medium (DMEM)/F-12 (1:1 mixture)	Corning	Cat# 10-090-CV
Dulbecco's phosphate-buffered saline (PBS)	Corning	Cat# 21-031-CV
E64	Goldbio	Cat# E-064-100
Fetal Calf Serum (FCS)	Sigma-Aldrich	Cat# F2442
FLAG peptide	Biomatik	Custom order
FreeStyle 293 Medium	Thermo Fisher	Cat# 12338018
Glyco-diosgenin (GDN)	Anatrace	Cat# GDN101
Lauryl Maltose Neopentyl Glycol (LMNG)	Anatrace	Cat# NG310
Leupeptin hemisulfate	Goldbio	Cat# L-010-100
Methyl- β-cyclodextrin (MCD)/Cholesterol complexes	Brown et. al., 2002(10)	N/A
NEBuilder HiFi DNA Assembly	NEB	at# E2621
Newborn calf lipoprotein-deficient serum (LPDS)	Goldstein et al., 1983(26)	N/A
Ni-NTA agarose beads	QIAGEN	Cat# 30250
Ni-NTA sepharose beads	Cytiva	Cat# 17531804
Opti-MEM	Thermo Fisher	Cat# 31985070
Penicillin-Streptomycin	Sigma-Aldrich	Cat# P4333
Pepstatin A	Santa Cruz	Cat# sc-45036B

Phenylmethylsulfonylfluoride (PMSF)	Goldbio	Cat# P-470-25
Platinum SuperFi II Master Mix	Thermo Fisher Scientific	Cat# 12368010
Protein A/G PLUS-Agarose	Santa Cruz Biotechnology	Cat# sc-2003
SF900 III SFM	Gibco	Cat# 12658019
Sodium butyrate	Sigma-Aldrich	Cat# 303410
Sodium cholate	Sigma-Aldrich	Cat# C1254
Sodium compactin	Brown et al., 1978(27)	N/A
Sodium mevalonate	Brown et al., 1978(27)	N/A
Sodium oleate	Goldstein et al., 1983(26)	N/A
SR-12813	Sever et al., 2004(28)	N/A
SuperSignal Substrate	Thermo Fisher	Cat# 34580
Trans-blot Turbo Nitrocellulose Transfer Pack	BioRad	Cat# 1704158
Tris (2-Carboxyethyl) phosphine Hydrochloride (TCEP-HCl)	Goldbio	Cat# TCEP1
X-tremeGENE HP DNA transfection reagent	Millipore Sigma	Cat# XTGHP-RO
**Critical commercial assays**		
Wizard® Plus SV Minipreps DNA Purification System	Promega	Cat# A1330
NucleoBond Xtra Midi	Macherey-Nagel	Cat# 740410
LookOut® Mycoplasma PCR Detection Kit	Millipore Sigma	Cat# MP0035
**Deposited data**		
PDB model of Scap^{EM}(TM1-8)/Insig-2/4G10^{Fab}	This paper	PDB: 9PXB
PDB model of Scap^{EM}(TM1-8)/Insig-2/4G10^{Fab} L1-L7 domain	This paper	PDB: 9PY6
PDB model of Scap^{EM}/4G10^{Fab} L1-L7 domain	This paper	PDB: 9PY7
Global map of Scap^{EM}(TM1-8)/Insig-2/4G10^{Fab}	This paper	EMD-71964
Focused refinement map of Scap^{EM}(TM1-8)/Insig-2/4G10^{Fab} L1-L7 domain	This paper	EMD-72010
Global map of Scap^{EM}/4G10^{Fab}	This paper	EMD-72029
Focused refinement map of Scap^{EM}/4G10^{Fab} L1-L7 domain	This paper	EMD-72012
**Experimental models: cell lines**		
Human: HEK293S GnTI^{-} cells	ATCC	Cat# CRL-3022
Hamster: Scap-deficient SRD-13A cells	Rawson et al., 1999(2)	N/A
Hamster: Insig-deficient SRD-15 cells	Lee et al., 2005(3)	N/A
Spodoptera frugiperda: Sf9 cells	Invitrogen	Cat# 11496015
**Recombinant DNA plasmids**		
pEZT-His$_{10}$-Scap^{WT}(L1-L7)	This paper	N/A
pEZT-His$_{10}$-Scap^{EM}(L1-L7)	This paper	N/A
pEZT-His$_{8}$-Scap^{WT}-3xFLAG	This paper	N/A
pEZT-His$_{8}$-Scap$^{I348A/Y351A/F417A}$-3xFLAG	This paper	N/A
pEZT-His$_{8}$-Scap$^{I348W/Y351W/F417W}$-3xFLAG	This paper	N/A
pEZT-His$_{8}$-Scap^{EM}-3xFLAG	This paper	N/A
pEZT-3xFLAG-TEV-His$_{8}$-Scap^{EM}(TM1-8)	This paper	N/A
pEZT-Insig-2	This paper	N/A
pEZT-4G10^{Fab}-His$_{10}$	This paper	N/A
pTK-3xFLAG-SREBP2	Kober et al. 2020(24)	N/A
pTK-Insig-2-6xMyc	Lee et al., 2006(9)	N/A
pTK-3xHA-Scap	This paper	N/A
pTK-Scap	Nohturfft et al., 1998(7)	N/A

pTK-Scap^{EM}	This paper	N/A
pTK-Scap Y298C	Yabe et al., 2002(29)	N/A
pTK-Scap D428A	Feramisco et al., 2005(30)	N/A
pTK-Scap L343A	This paper	N/A
pTK-Scap L343W	This paper	N/A
pTK-Scap N344A	This paper	N/A
pTK-Scap N344W	This paper	N/A
pTK-Scap E347A	This paper	N/A
pTK-Scap E347W	This paper	N/A
pTK-Scap I348A	This paper	N/A
pTK-Scap I348A/Y351A/F417A	This paper	N/A
pTK-Scap I348W	This paper	N/A
pTK-Scap I348W/Y351W/F417W	This paper	N/A
pTK-Scap Y351A	This paper	N/A
pTK-Scap Y351W	This paper	N/A
pTK-Scap L352C/I520C	This paper	N/A
pTK-Scap V354A	This paper	N/A
pTK-Scap V354W	This paper	N/A
pTK-Scap V355A	This paper	N/A
pTK-Scap V355W	This paper	N/A
pTK-Scap V355C/I520C	This paper	N/A
pTK-Scap I356C/I520C	This paper	N/A
pTK-Scap L362C/A511C	This paper	N/A
pTK-Scap E399C/Q517C	This paper	N/A
pTK-Scap E399C/M521C	This paper	N/A
pTK-Scap I402A	This paper	N/A
pTK-Scap I402W	This paper	N/A
pTK-Scap I403A	This paper	N/A
pTK-Scap I403W	This paper	N/A
pTK-Scap L410C/L539C	This paper	N/A
pTK-Scap L410C/Y542C	This paper	N/A
pTK-Scap L410C/Y543C	This paper	N/A
pTK-Scap I414A	This paper	N/A
pTK-Scap I414A/L531A	This paper	N/A
pTK-Scap I414C	This paper	N/A
pTK-Scap I414W	This paper	N/A
pTK-Scap I414C/I528C	This paper	N/A
pTK-Scap I414C/L531C	This paper	N/A
pTK-Scap F417A	This paper	N/A
pTK-Scap F417W	This paper	N/A
pTK-Scap I520A	This paper	N/A
pTK-Scap I520W	This paper	N/A
pTK-Scap I520W/W527A	This paper	N/A
pTK-Scap M521A	This paper	N/A
pTK-Scap T524A	This paper	N/A
pTK-Scap W527A	This paper	N/A
pTK-Scap I528A	This paper	N/A
pTK-Scap I528W	This paper	N/A
pTK-Scap L531A	This paper	N/A
pTK-Scap L531C	This paper	N/A
pTK-Insig-2-6xMyc I118A	This paper	N/A
pTK-Insig-2-6xMyc I118W	This paper	N/A
pTK-Insig-2-6xMyc H120A	This paper	N/A

pTK-Insig-2-6xMyc H120W	This paper	N/A
pTK-Insig-2-6xMyc A121W	This paper	N/A
pTK-Insig-2-6xMyc K124A	This paper	N/A
pTK-Insig-2-6xMyc K124W	This paper	N/A
pTK-Insig-2-6xMyc L140A	This paper	N/A
**Software and algorithms**		
AlphaFold2 v2.0	Jumper et al., 2021(31)	https://alphafold.ebi.ac.uk/
CryoSPARC v3.5.2	Punjani et al., 2017(11)	https://cryosparc.com/
DeepEMhancer v0.14	Sanchez-Garcia et al., 2021(17)	https://github.com/rsanchezgarc/deepEMhancer
Fiji 2.16.0	National Institutes of Health	https://imagej.net/software/fiji/
Gctf	Zhang, 2016(13)	https://www.mrc-lmb.cam.ac.uk/kzhang/Gctf/
Illustrator 2025	Adobe	https://www.adobe.com/products/illustrator.html
ISOLDE 1.6	Croll, 2018(21)	https://tristanic.github.io/isolde/index.html
Molprobity 4.5.2	Chen et al., 2010(22)	http://molprobity.biochem.duke.edu/
MotionCor2.1	Zheng et al., 2017(12)	https://emcore.ucsf.edu/ucsf-motioncor2
Phenix (1.20.1-4487)	Liebschner et al., 2019(20)	https://phenix-online.org/download/
Prism 10	GraphPad	https://www.graphpad.com/scientificsoftware/prism/
PyMOL (2.5.2)	Schrödinger	https://pymol.org/
Relion 3.1	Zivanov et al., 2018(14)	https://www3.mrc-lmb.cam.ac.uk/ relion/index.php/Main_Page
Relion 5.0	Kimanius et al., 2024(16)	https://www3.mrc-lmb.cam.ac.uk/ relion/index.php/Main_Page
SerialEM 3.9	Mastronarde, 2005(32)	https://bio3d.colorado.edu/SerialEM/
UCSF ChimeraX 1.6.1	Pettersen et al., 2021(23)	https://www.cgl.ucsf.edu/chimerax/
WinCoot 0.9.8.7	Emsley et al., 2010(19)	https://www2.mrc-lmb.cam.ac.uk/ personal/pemsley/coot/
wwPDB Validation System V5.33/0.58	Burley et al., 2017(33)	https://validate-rcsb-1.wwpdb.org/
**Other**		
Quantifoil® Holey Carbon R 1.2/1.3 300 mesh Au grids	Ted Pella	Cat# 658-300-AU
Superose^{TM} 6 Increase 10/300 column	GE Healthcare	Cat# 29091596
Superdex^{TM} 200 Increase 10/300 column	GE Healthcare	Cat# 28990944

Figure S1

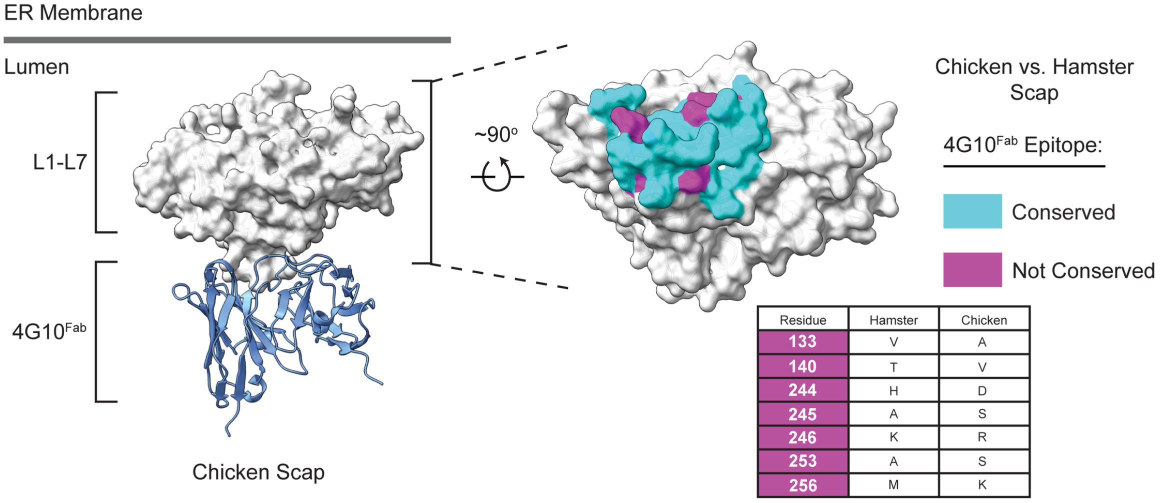

Residue	Hamster	Chicken
133	V	A
140	T	V
244	H	D
245	A	S
246	K	R
253	A	S
256	M	K

Fig. S1. Comparison of the 4G10^{Fab}-binding region of chicken Scap to the corresponding region of hamster Scap.

(*Left*) Space filling model of chicken Scap(L1-L7) in grey bound to 4G10^{Fab} (blue; PDB: 7LKH) in the ER lumen. (*Right*) The model from the left was rotated ~90°, zooming in on where 4G10^{Fab} interacts with the L1-L7 domain. At the interaction site, the space filling model of each residue is colored relative to whether the residue is conserved between chicken Scap (XP_046768602.1) and hamster Scap (P97260.1). Conserved residues are colored in cyan and residues that are not conserved are colored in magenta (summarized in table).

Figure S2

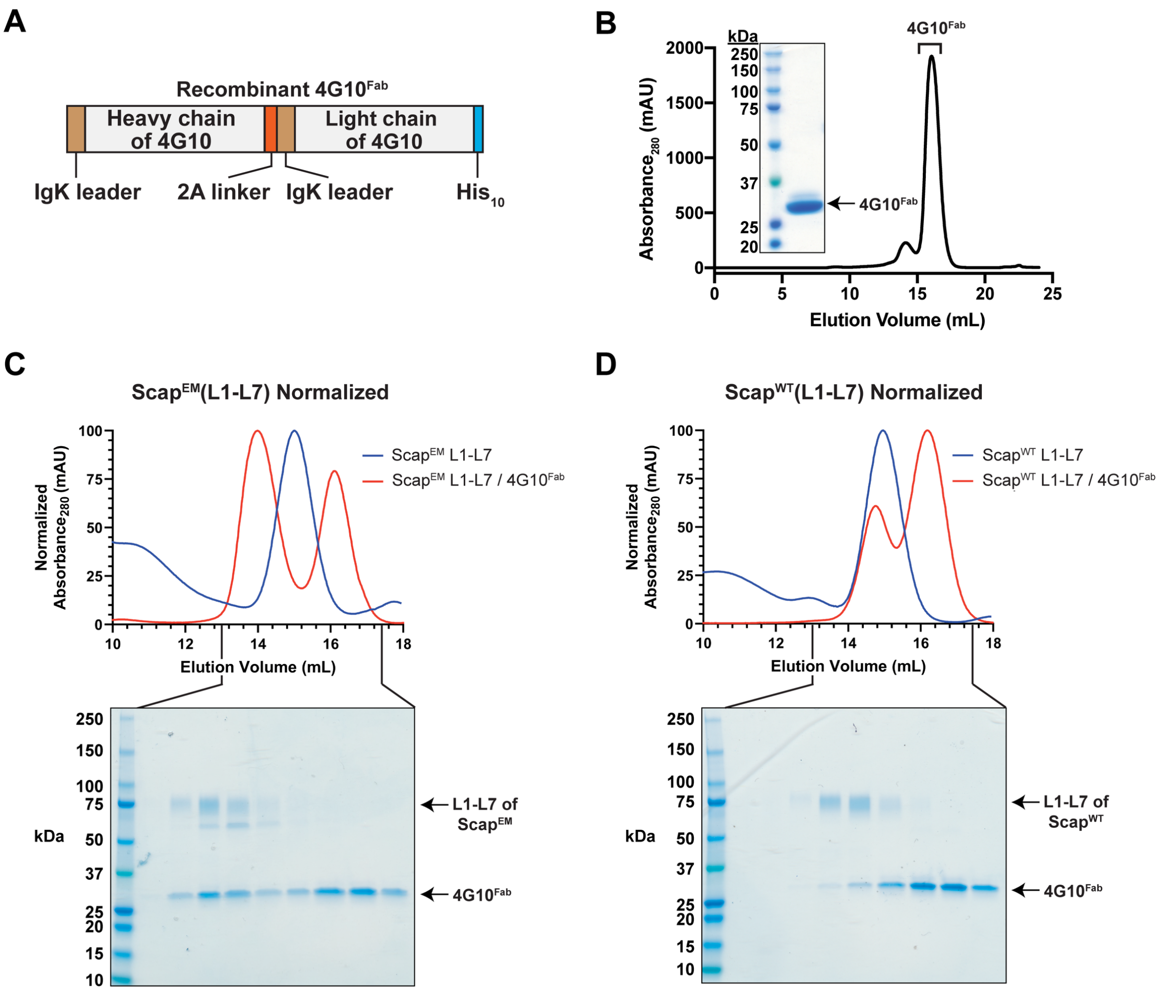

Fig. S2. Biochemical properties of recombinant $4G10^{Fab}$.

(*A*) Schematic of plasmid encoding $4G10^{Fab}$. The full sequence details are listed in **Methods**. (*B*) Purification of recombinant $4G10^{Fab}$. Shown is a representative gel filtration chromatography trace of $4G10^{Fab}$ purified as described in **Methods**. (*Inset*) An aliquot of the peak fraction was subjected to SDS-PAGE followed by Coomassie Blue staining. (*C*, *D*) Comparison of binding of $4G10^{Fab}$ to hamster $Scap^{EM}$(L1-L7) and hamster $Scap^{WT}$(L1-L7). Binding between $4G10^{Fab}$ and Scap proteins was assessed as described in **Methods**. Gel filtration chromatography traces (*top panels*) are shown along with SDS-PAGE of the indicated fractions followed by Coomassie Blue staining (*bottom panels*).

Figure S3

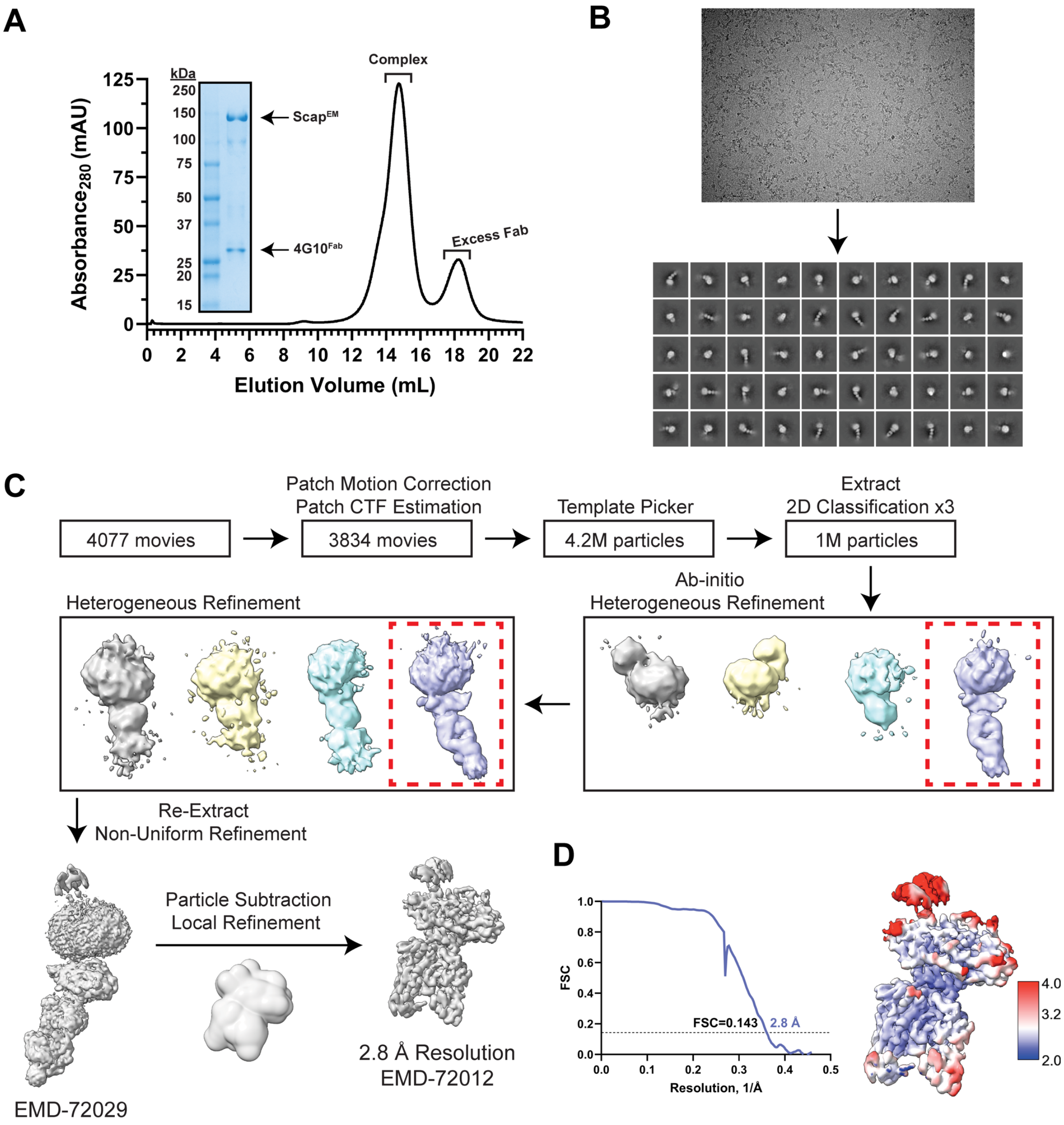

Fig. S3. Cryo-EM analysis of Scap^{EM}/4G10^{Fab}.

(*A*) Purification of Scap^{EM}/4G10^{Fab} complexes. Purified Scap^{EM} was incubated with 1.5-fold molar excess of freshly purified 4G10^{Fab} for 30 min on ice and then subjected to gel filtration chromatography as described in **Methods**. An aliquot of the peak fraction was subjected to 4-15% SDS-PAGE followed by Coomassie Blue staining. (*B* – *D*) Cryo-EM workflow. (*B*) Representative electron micrograph and 2D class averages of Scap^{EM}/4G10^{Fab}. (*C*) Flowchart of cryo-EM data processing steps that yielded both the full 3D reconstruction as well as a focused refinement map of the L1-L7 domain bound to 4G10^{Fab}. (*D*) Fourier shell correlation (FSC) curve for the cryo-EM reconstruction focused on the L1-L7 domain bound to 4G10^{Fab} (*left*) along with this reconstruction colored by local resolution (*right*).

Figure S4

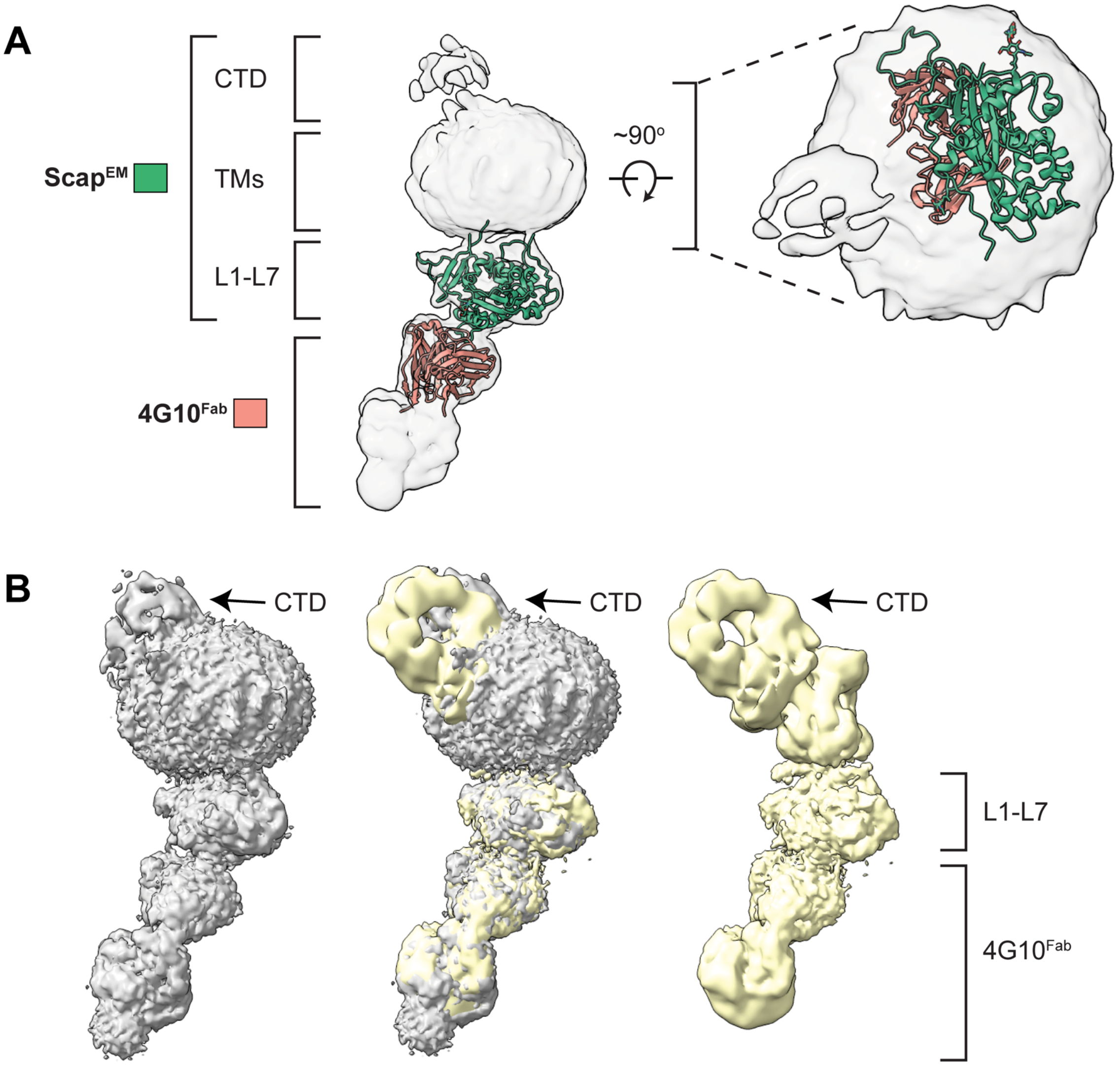

Fig. S4. Relative orientations of Scap's membrane domain and L1-L7 domains.

(*A*) Cryo-EM reconstruction of Scap^{EM}/4G10^{Fab}. After Gaussian-filtering to 1.5 standard deviations, the map was contoured to 7.5 σ, colored transparent white and fit with the cartoon model of Scap^{EM}(L1-L7)/4G10^{Fab} in ChimeraX. In the cartoon model, 4G10^{Fab} was colored light pink and Scap^{EM}(L1-L7) was colored green. (*Inset*) Zoomed-in view of the transmembrane (TM) region from above, which shows that the L1-L7 domain is oriented beneath the micelle. *CTD*, C-terminal domain of Scap. (*B*) Comparison of structures. (*Left*) Cryo-EM reconstruction of Scap^{EM}/4G10^{Fab} contoured to 5σ (shaded gray in ChimeraX). (*Right*) Cryo-EM reconstruction of chicken Scap/4G10^{Fab} (EMD-23406) contoured to 5σ (shaded light yellow in ChimeraX). (*Middle*) Overlay of the two structures. A map-in-map fit using ChimeraX resulted in a correlation value of 0.941. Both reconstructions feature L1-L7 domains directly underneath their corresponding micelles and a ring-like feature (CTD) protruding from similar regions of each micelle.

Figure S5

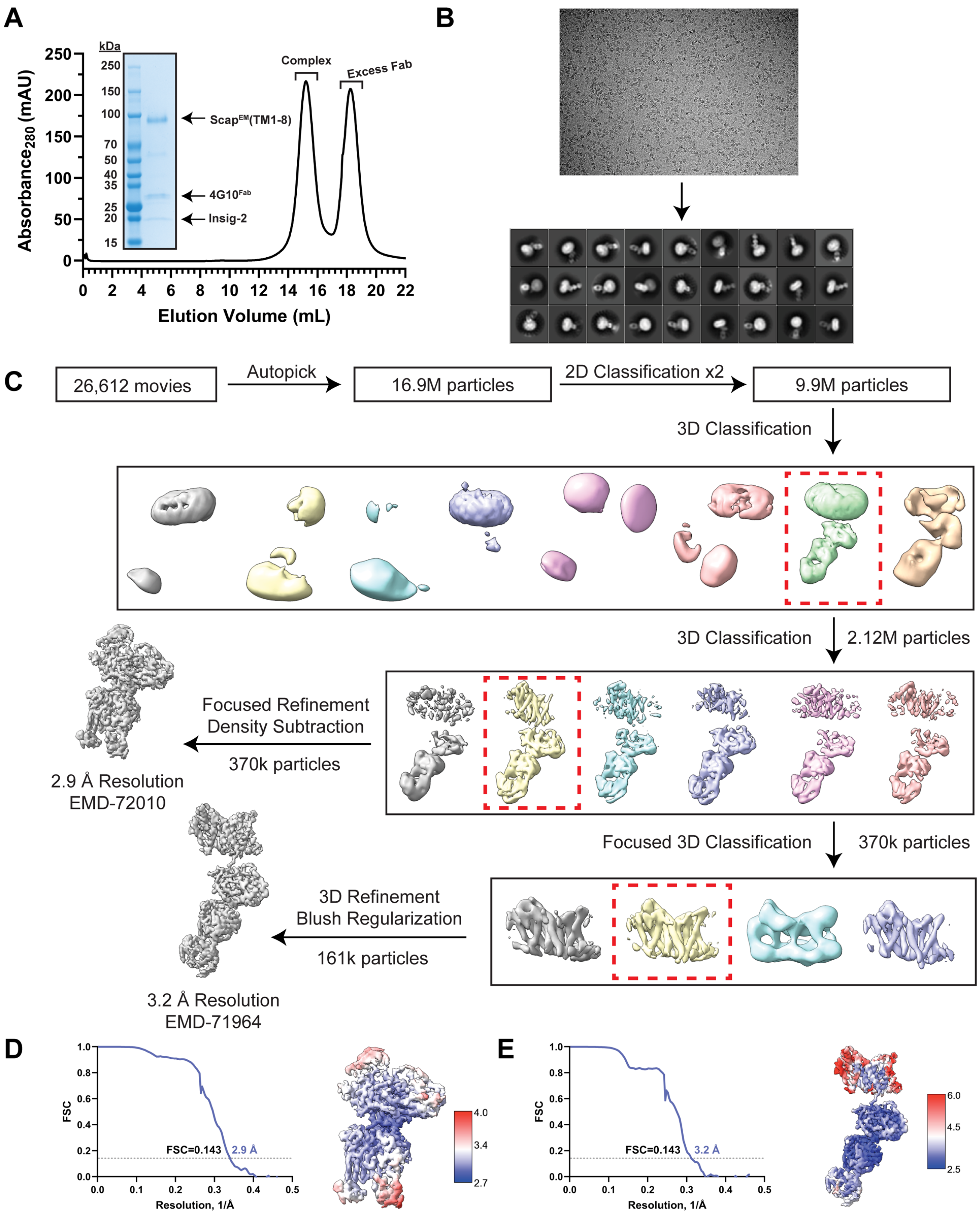

Fig. S5. Cryo-EM analysis of Scap^{EM}(TM1-8)/Insig-2/4G10^{Fab}.

(*A*) Purification of Scap^{EM}(TM1-8)/Insig-2/4G10^{Fab} complexes. Purified Scap^{EM}(TM1-8) was incubated with 3-fold molar excess of 4G10^{Fab} (thawed from a -80°C stock) for 30 min on ice and then subjected to gel filtration chromatography as described in **Methods**. An

aliquot of the peak fraction was subjected to 4-15% SDS-PAGE followed by Coomassie Blue staining. (*B* – *D*) Cryo-EM workflow. (*B*) Representative electron micrograph and 2D class averages of $Scap^{EM}$(TM1-8)/Insig-2/$4G10^{Fab}$. (*C*) Flowchart of cryo-EM data processing steps that yielded both the full 3D reconstruction as well as a focused refinement map of the L1-L7 domain bound to $4G10^{Fab}$. (*D*) Fourier shell correlation (FSC) curve for the cryo-EM reconstruction focused on the L1-L7 domain bound to $4G10^{Fab}$ (*left*) along with this reconstruction colored by local resolution (*right*). (*E*) FSC curve for the full cryo-EM reconstruction (*left*) along with this reconstruction colored by local resolution (*right*).

Figure S6

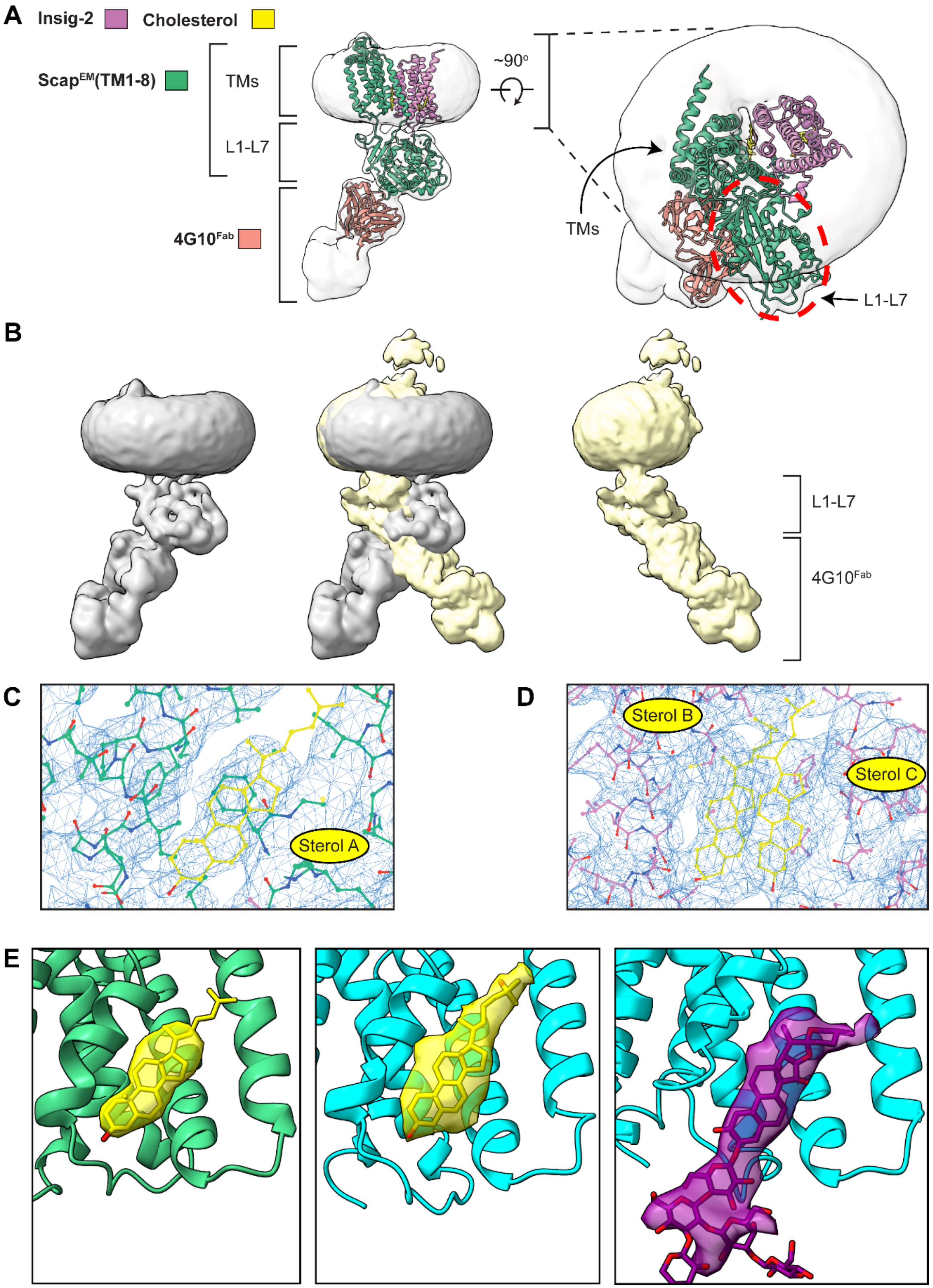
A
Insig-2
Cholesterol
Scap^EM(TM1-8)
TMs
L1-L7
4G10^Fab
~90°
TMs
L1-L7
B
L1-L7
4G10^Fab
C
Sterol A
D
Sterol B
Sterol C
E

Fig. S6. Comparisons of structures of Scap^{EM}(TM1-8)/Insig-2/4G10^{Fab} to Scap^{EM}/4G10^{Fab} and analysis of bound ligand densities.

(*A*) Cryo-EM reconstruction of Scap^{EM}(TM1-8)/Insig-2/4G10^{Fab}. After Gaussian-filtering to 1.5 standard deviations, the map was contoured to 5 σ, colored transparent white and fit with the cartoon model of Scap^{EM}(TM1-8)/Insig-2/4G10^{Fab} in ChimeraX. In the cartoon model, 4G10^{Fab} was colored light pink, Scap^{EM}(TM1-8) was colored green, and Insig-2 was colored magenta. (*Inset*) Zoomed-in view of the TM region from above, which shows that the L1-L7 domain (*red dashed outline*) is oriented not beneath the TMs, but rather is rotated outwards. (*B*) Comparison of structures. (*Left*) Cryo-EM reconstruction of Scap^{EM}(TM1-8)/Insig-2/4G10^{Fab}, Gaussian-filtered to 1.5 standard deviations, and contoured to 5σ (shaded gray in ChimeraX). (*Right*) Cryo-EM reconstruction of Scap^{EM}/4G10^{Fab}, Gaussian-filtered to 1.5 standard deviations, contoured to 7.5σ (shaded light yellow in ChimeraX), and oriented by an alignment based on a homology model of chicken Scap aligned with Scap^{EM}(TM1-8)/Insig-2/4G10^{Fab} by their TM regions in PyMOL. The reconstruction of Scap^{EM}/4G10^{Fab} was then fitted to the aligned chicken Scap model in ChimeraX. (*Middle*) Overlay of the two maps. Aligning the two maps by their fitted models in the TM region highlights that the L1-L7 domains in the free Scap (*light yellow*) and the Insig-bound Scap (*gray*) are facing in opposite directions. (*C*, *D*) Modeling of sterols in the Scap^{EM}(TM1-8)/Insig-2/4G10^{Fab} reconstruction. Shown is the cryo-EM reconstruction of Scap^{EM}(TM1-8)/Insig-2/4G10^{Fab} with its docked model. The reconstruction was contoured to 5σ and represented as blue-colored mesh in ChimeraX. The model is shown as balls and sticks with Scap colored in green, Insig-2 colored in magenta, and cholesterol colored in yellow. (*C*) Zoomed-in view of the Scap/Insig-2 interface from the perspective of Insig-2 shows cholesterol (sterol A) interacting with Scap residues. (*D*) Zoomed-in view of the TM region of Insig-2 showing cholesterols (sterol B on left and sterol C on right) filling a cavity. (*E*) Comparison of molecules bound to Scap in published structures. Models for hamster Scap^{EM}(TM1-8)/Insig-2 bound to cholesterol (*left, green,* PDB:9PXB, current study), human Scap(TM1-8)/Insig-2 bound to 25-hydroxycholesterol (*middle, cyan*, PDB:6M49), and human Scap(TM1-8)/Insig-2 bound to digitonin (*right, cyan,* PDB:7ETW) were aligned by TMs 1-6 in PyMOL and oriented facing the Sterol A site (current study). For sake of clarity, we excluded the models for Insigs in this view. Cryo-EM reconstructions of each complex (EMD-71964, EMD-30074, and EMD-31303 respectively) were contoured to 5σ, fit to their corresponding model, and colored using colorzone in ChimeraX with cholesterol and 25-hydroxycholesterol colored in yellow and digitonin colored in purple.

Figure S7

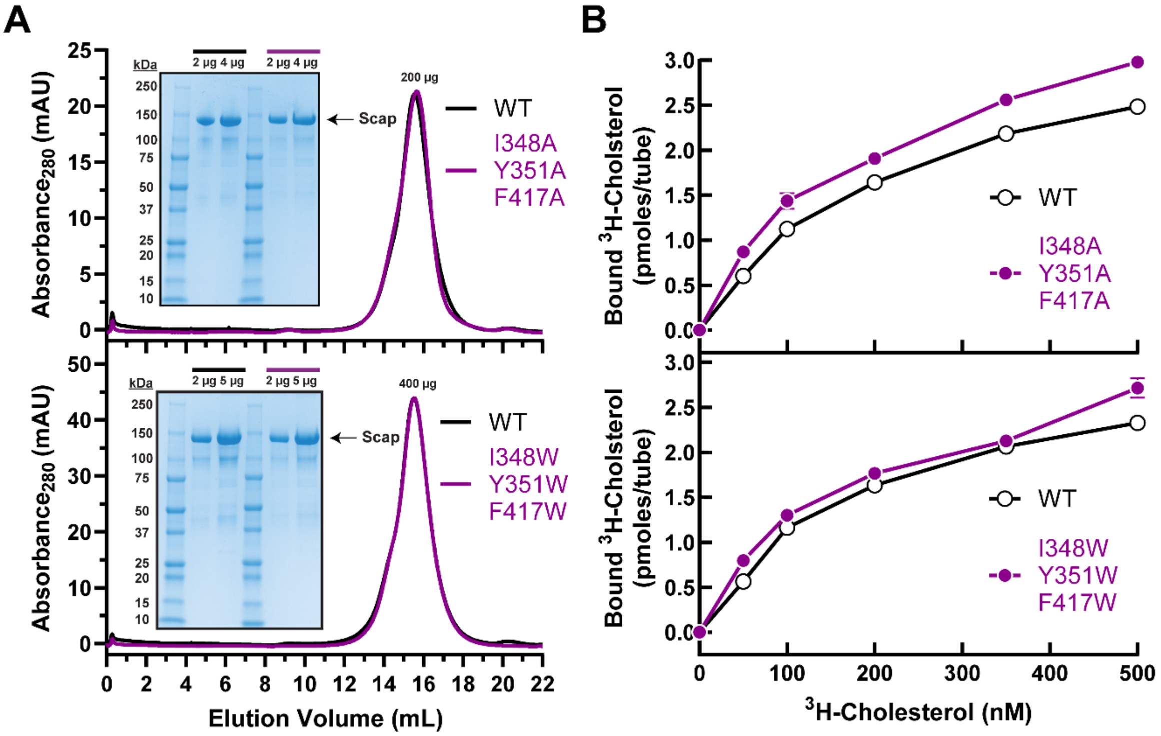

Fig. S7. Comparison of ^{3}H-cholesterol binding to WT and mutant Scaps.

Comparison of biochemical properties of wild-type (WT) and triple mutant versions of Scap. (*A*) Gel filtration chromatography. WT and indicated triple mutant versions of Scap were purified as described in **Methods** and aliquots of the purified proteins (200 µg in *top panel*, 400 µg in *bottom panel*) were subjected to gel filtration chromatography. (*Insets*) Aliquots (2 or 4 µg in *top panel*, 2 or 5 µg in *bottom panel*) of each protein were subjected to 4-15% SDS-PAGE followed by Coomassie Blue staining. (*B*) Cholesterol binding. Each binding reaction contained 2 µg of WT or the indicated triple mutant versions of Scap and varying concentrations of ^{3}H-cholesterol (133,200 dpm/pmol). After incubation for 4 h at 4°C, cholesterol binding was measured as described in **Methods**. Each data point denotes the average of three replicates, and error bars represent the standard error. When not visible, error bars are smaller than the size of the symbols.

Figure S8

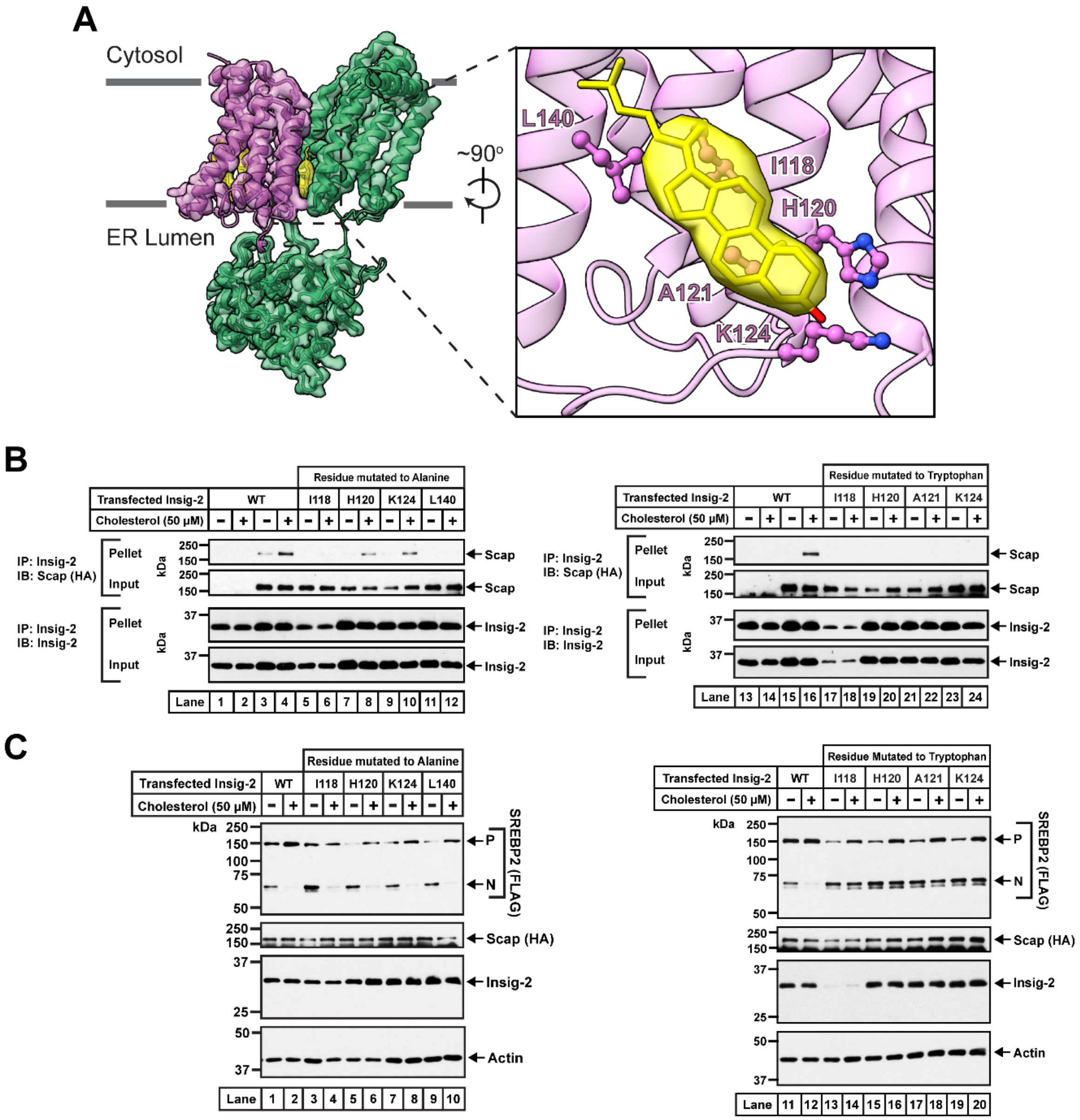

Fig. S8. Mutational analysis of Insig-2 residues at the Scap/cholesterol/Insig-2 interface.

(*A*) Structural model. Shown is the model and cryo-EM reconstruction of $Scap^{EM}$(TM1-8) (*green*) bound to Insig-2 (*magenta*) with associated sterols (*yellow*) (same as in **Fig. 3A**, *left panel*). (*Inset*) A zoomed-in view of this interface focusing on Insig-2 and sterol A. Sidechains of five Insig-2 residues in close proximity of sterol A are shown as ball and stick models. (*B*) Binding of Insig-2 mutants to Scap. Insig-1/Insig-2-deficient hamster SRD-15 cells were set up and transfected as described in **Methods** with 2 μg of pTK-3xHA-Scap and 4 μg of either WT of indicated mutant versions of pTK-Insig-2-6xMyc. The control conditions in *lanes 1* and *13* were transfected with 2 μg of pTK-Insig-2-6xMyc and 4 μg of pcDNA3 empty vector plasmid. The transfected cells were depleted of cholesterol

and replenished with the indicated concentrations of cholesterol (delivered in complexes with MCD), after which the cells were harvested and processed for co-immunoprecipitation assays and immunoblot analysis as described in **Methods**. (*C*) Ability of Insig-2 mutants to regulate cleavage of SREBP2. Insig-1/Insig-2-deficient hamster SRD-15 cells were set up and transfected as described in **Methods** with 2 µg of pTK-3xFLAG-SREBP2, 2 µg of pTK-3xHA-Scap, and 3 µg of either WT or indicated mutant versions of pTK-Insig-2-6xMyc. The transfected cells were then depleted of cholesterol and replenished with the indicated concentrations of cholesterol (delivered in complexes with MCD), after which cell lysates were analyzed by immunoblot as described in **Methods**. *P*, precursor form of SREBP2; *N*, cleaved nuclear form of SREBP2.

Figure S9

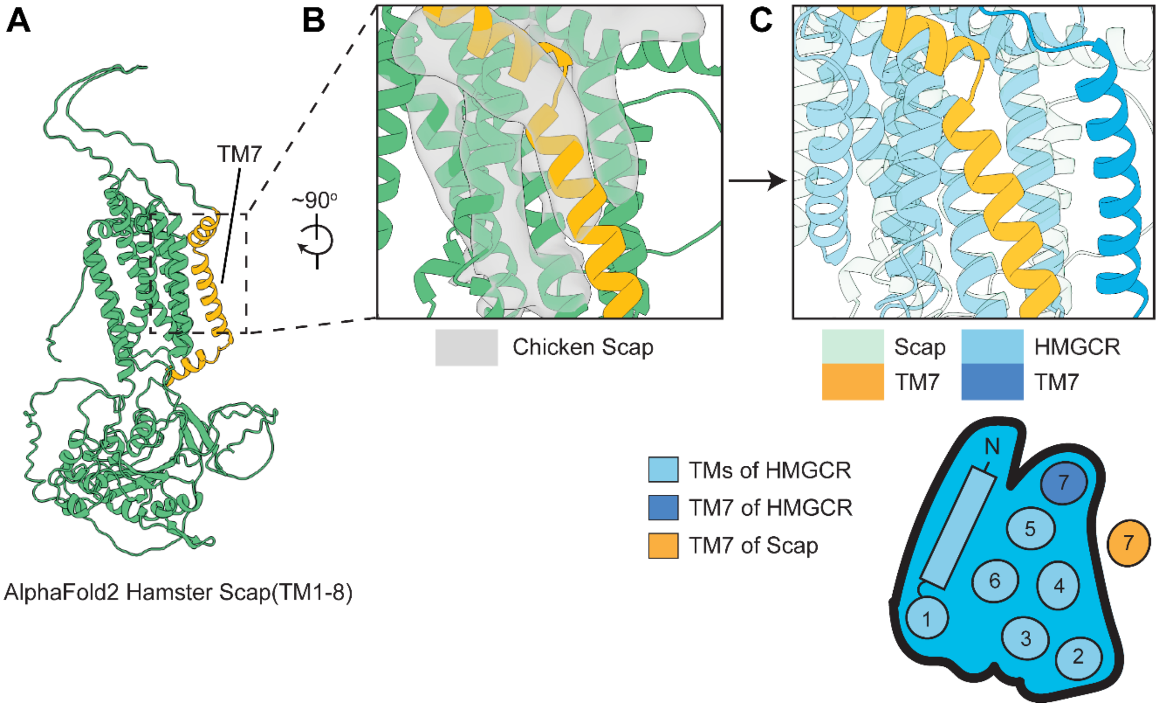

Fig. S9. Comparisons of the positioning of TM7 in Scap and HMG-CoA reductase (HMGCR).

(*A*) Model of hamster Scap. Shown is a cartoon model predicted by AlphaFold2 of the membrane domain of hamster Scap (aa 1-752) [AF-P97260-F1-v4]. For sake of comparison, the model is shown in the same orientation as Scap when it is complexed to Insig-2 complex (**Fig. 2B**, *left panel*). The predicted Scap model is colored green except for TM7 which is colored orange. (*B*) Zoomed-in view of the TM7 region of Scap. The model in *A* was rotated ~90° clockwise relative to the center, zooming in on the region where TM7 interacts with the rest of the transmembrane region. This model is fitted into our previous cryo-EM reconstruction of chicken Scap (EMD-23406) aligned by the transmembrane region and contoured to 8.5σ using ChimeraX (*gray*). (*C*) Comparison of TM architectures of Scap and HMGCR. (*Top*) Shown is a structural model of HMGCR complexed to UbiA Prenyltransferase Domain-Containing Protein-1 (UBIAD1) in conformation B (PDB:8DJK) overlaid on a transparent Scap model (with the exception of TM7) from *B*. The structures are aligned by TMs 2-6 on HMGCR and Scap. For clarity, UBIAD1 is omitted. HMGCR is colored in light blue, except for its TM7 which is colored dark blue. (*Bottom*) Shown is a model outline of the cytosolic membrane view of HMGCR. HMGCR's TM7 is packed against its TM5 in a position near where Scap's TM7, shown in orange, packs against Scap's TM4/TM5/TM6 surface.

Figure S10

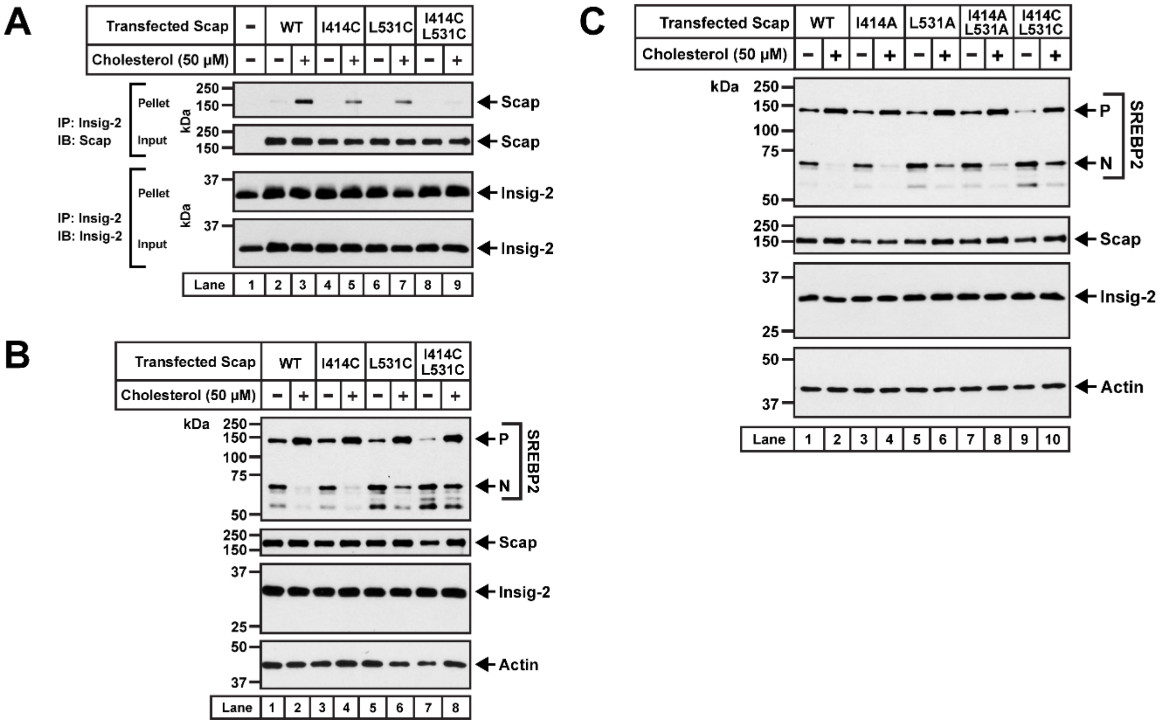

Fig. S10. Mutational analysis of two amino acid residues (I414 and L531) on Scap.

(*A*) Binding of Scap mutants to Insig-2. Scap-deficient SRD-13A cells were set up and transfected as described in **Methods** with 2 µg of pTK-Insig-2-6xMyc and 4 µg of either WT or indicated mutant versions of pTK-Scap. The control condition in *lane 1* was transfected with 2 µg of pTK-Insig-2-6xMyc and 4 µg of pcDNA3 empty vector plasmid. The transfected cells were then depleted of cholesterol and replenished with the indicated concentrations of cholesterol (delivered in complexes with MCD), after which the cells were harvested and processed for co-immunoprecipitation assays and immunoblot analysis as described in **Methods**. (*B* – *C*) Ability of Scap mutants to mediate cleavage of SREBP2. Scap-deficient SRD-13A cells were set up and transfected as described in **Methods** with 2 µg of pTK-3xFLAG-SREBP2, 1.5 µg of pTK-Insig-2-6xMyc, and 2 µg of either WT or indicated mutant versions of pTK-Scap. The transfected cells were then depleted of cholesterol and replenished with the indicated concentrations of cholesterol (delivered in complexes with MCD), after which cell lysates were analyzed by immunoblot as described in **Methods**. *P*, precursor form of SREBP2; *N*, cleaved nuclear form of SREBP2.

Tables

Table S1. Cryo-EM data collection, refinement, and validation statistics.

	Scap^{EM}/Insig-2/4G10^{Fab} (EMD-71964) (PDB 9PXB)	Scap^{EM}/Insig-2/4G10^{Fab} (L1L7) (EMD-72010) (PDB 9PY6)	Scap^{EM}/4G10^{Fab} (EMD-72029)	Scap^{EM}/4G10^{Fab} (L1L7) (EMD-72012) (PDB 9PY7)
**Data collection and processing**				
Magnification	46,296		46,296	
Voltage (kV)	300		300	
Electron exposure (e–/Å^2)	60		60	
Defocus range (μm)	-1.4 to -2.4		-1.4 to -2.4	
Pixel size (Å)	1.07		1.0748	
Symmetry imposed	C1		C1	
Initial particle images (no.)	16,886,990		2,896,240	
Final particle images (no.)	160,849	370,255	272,072	
Map resolution (Å)<br>FSC threshold	3.2<br>0.143	2.9<br>0.143	3.0<br>0.143	2.8<br>0.143
Map resolution range (Å)	n/a	n/a	n/a	n/a
**Refinement**				
Initial model used (PDB code)	7ETW	7LKH		7LKF
Model resolution (Å)<br>FSC threshold	3.4<br>0.5	3.1<br>0.5		2.9<br>0.5
Model resolution range (Å)	n/a	n/a		n/a
Map sharpening *B* factor (Å^2)	n/a	n/a		n/a
Model composition<br>Non-hydrogen atoms<br>Protein residues<br>Ligands	 7638<br>950<br>5	 4334<br>537<br>2		 4348<br>537<br>3
B factors (Å^2)<br>Protein<br>Ligand	 n/a<br>n/a	 n/a<br>n/a		 n/a<br>n/a
R.m.s. deviations<br>Bond lengths (Å)<br>Bond angles (°)	 0.002<br>0.527	 0.002<br>0.518		 0.003<br>0.530
Validation<br>MolProbity score<br>Clashscore<br>Poor rotamers (%)	 1.60<br>6.72<br>0.00	 1.21<br>2.69<br>0.00		 1.34<br>4.31<br>0.00
Ramachandran plot<br>Favored (%)<br>Allowed (%)<br>Disallowed (%)	 96.49<br>3.51<br>0.00	 97.16<br>2.84<br>0.00		 97.35<br>2.65<br>0.00
Protein residues included in the model	Scap: 2-451, 609-684	Scap: 52-282, 609-684		Scap: 52-282, 609-684
	Insig-2: 21-214			
	4G10^{Fab}: HC: 6-126, LC: 6-114	4G10^{Fab}: HC: 6-126, LC: 6-114		4G10^{Fab}: HC: 6-126, LC: 6-114

Table S2. Functional characterization of Scap mutants.

Scap Mutation(s)	Location on Scap	Transports to Golgi		Binds Insig-2	
		Cholesterol		Cholesterol	
		Low	High	Low	High
WT		+	-	-	+
Y298C	TM2	+	+	-	-
D428A	TM6	-	-	+	+
V133A;T140V;H244D;A245S; K246R;A253S;M256K	Loop1	+	-	NT	NT
L343A	Loop3	+	-	-	+
L343W	Loop3	+	-	-	+
N344A	Loop3	+	-	-	+
N344W	Loop3	+	-	-	+
E347A	TM4	+	-	-	+
E347W	TM4	+	+	-	-
I348A	TM4	+	-	-	+
I348A;Y351A;F417A	TM4;TM6	-	-	-	-
I348W	TM4	+	-	-	-
I348W;Y351W;F417W	TM4;TM6	-	-	-	-
Y351A	TM4	-	-	-	-
Y351W	TM4	+	-	-	+
L352C;I520C	TM4;TM7	NT	-	NT	NT
V354A	TM4	+	-	-	+
V354W	TM4	+	-	-	-
V355A	TM4	+	+	-	+
V355C;I520C	TM4;TM7	NT	-	NT	NT
V355W	TM4	+	-	-	+
I356C;I520C	TM4;TM7	NT	-	NT	NT
L362C;A511C	TM4;TM7	NT	-	NT	NT
E399C;Q517C	TM5;TM7	NT	-	NT	NT
I402A	TM5	-	-	+	+
I402W	TM5	-	-	-	+
I403A	TM5	+	-	-	+
I403W	TM5	-	-	+	+
L410C;L539C	TM5;TM7	NT	-	NT	NT
L410C;Y542C	TM5;TM7	NT	-	NT	NT
L410C;Y543C	TM5;TM7	NT	-	NT	NT
I414A	TM6	+	-	-	-
I414A;L531A	TM6;TM7	+	-	NT	NT
I414C	TM6	+	-	-	+
I414C;I528C	TM6;TM7	NT	-	NT	NT
I414C;L531C	TM6;TM7	+	+	-	-
I414W	TM6	-	-	-	-
F417A	TM6	-	-	-	-
F417W	TM6	-	-	-	-
I520A	TM7	-	NT	NT	NT
I520W	TM7	-	NT	+	+
I520W;W527A	TM7	NT	NT	+	+
M521A	TM7	+	NT	NT	NT
T524A	TM7	+	NT	NT	NT
W527A	TM7	-	NT	+	+

I528A	TM7	+	NT	NT	NT
I528W	TM7	+	NT	NT	NT
L531A	TM7	+	-	NT	NT
L531C	TM7	+	-	-	+

NT – Not Tested, TM – Transmembrane helix

Table S3. Functional characterization of Insig-2 mutants.

Insig-2 Mutation	Location	Inhibits Scap Transport		Binds Scap	
		Cholesterol		Cholesterol	
		Low	High	Low	High
WT		-	+	-	+
I118A	TM3	-	+	-	-
I118W	TM3	NE	NE	NE	NE
H120A	TM3	-	+	-	+
H120W	TM3	-	-	-	-
A121W	TM3	-	-	-	-
K124A	TM3	-	+	-	+
K124W	TM3	-	-	-	-
L140A	TM4	-	+	-	-

NE – Not Expressed, TM – Transmembrane helix

SI References

1. D. L. Kober, *et al.*, Scap structures highlight key role for rotation of intertwined luminal loops in cholesterol sensing. *Cell* **184**, 3689-3701.e22 (2021).
2. R. B. Rawson, R. DeBose-Boyd, J. L. Goldstein, M. S. Brown, Failure to cleave sterol regulatory element-binding proteins (SREBPs) causes cholesterol auxotrophy in Chinese hamster ovary cells with genetic absence of SREBP cleavage-activating protein. *J Biol Chem* **274**, 28549–28556 (1999).
3. P. C. W. Lee, N. Sever, R. A. DeBose-Boyd, Isolation of sterol-resistant Chinese hamster ovary cells with genetic deficiencies in both Insig-1 and Insig-2. *J Biol Chem* **280**, 25242–25249 (2005).
4. Z. Liu, *et al.*, Systematic comparison of 2A peptides for cloning multi-genes in a polycistronic vector. *Sci Rep* **7**, 2193–9 (2017).
5. Y. Zhang, *et al.*, Direct Demonstration That Loop1 of Scap Binds to Loop7 A CRUCIAL EVENT IN CHOLESTEROL HOMEOSTASIS*. *J. Biol. Chem.* **291**, 12888–12896 (2016).
6. C. L. Morales-Perez, C. M. Noviello, R. E. Hibbs, Manipulation of Subunit Stoichiometry in Heteromeric Membrane Proteins. *Structure* **24**, 797–805 (2016).
7. A. Nohturfft, M. S. Brown, J. L. Goldstein, Sterols regulate processing of carbohydrate chains of wild-type SREBP cleavage-activating protein (SCAP), but not sterol-resistant mutants Y298C or D443N. *Proc Natl Acad Sci USA* **95**, 12848–12853 (1998).
8. X. Hua, J. Sakai, M. S. Brown, J. L. Goldstein, Regulated cleavage of sterol regulatory element binding proteins requires sequences on both sides of the endoplasmic reticulum membrane. *J Biol Chem* **271**, 10379–10384 (1996).
9. J. N. Lee, Y. Gong, X. Zhang, J. Ye, Proteasomal degradation of ubiquitinated Insig proteins is determined by serine residues flanking ubiquitinated lysines. *Proc. Natl. Acad. Sci.* **103**, 4958–4963 (2006).
10. A. J. Brown, L. Sun, J. D. Feramisco, M. S. Brown, J. L. Goldstein, Cholesterol addition to ER membranes alters conformation of SCAP, the SREBP escort protein that regulates cholesterol metabolism. *Mol Cell* **10**, 237–245 (2002).
11. A. Punjani, J. L. Rubinstein, D. J. Fleet, M. A. Brubaker, cryoSPARC: algorithms for rapid unsupervised cryo-EM structure determination. *Nat Methods* **14**, 290–296 (2017).
12. S. Q. Zheng, *et al.*, MotionCor2: anisotropic correction of beam-induced motion for improved cryo-electron microscopy. *Nat Meth* **14**, 331–332 (2017).
13. K. Zhang, Gctf: Real-time CTF determination and correction. *J Struct Biol* **193**, 1–12 (2016).
14. J. Zivanov, *et al.*, New tools for automated high-resolution cryo-EM structure determination in RELION-3. *Elife* **7**, 163 (2018).
15. X.-C. Bai, E. Rajendra, G. Yang, Y. Shi, S. H. W. Scheres, Sampling the conformational space of the catalytic subunit of human γ-secretase. *Elife* **4**, 1485 (2015).
16. D. Kimanius, *et al.*, Data-driven regularization lowers the size barrier of cryo-EM structure determination. *Nat. Methods* **21**, 1216–1221 (2024).
17. R. Sanchez-Garcia, *et al.*, DeepEMhancer: a deep learning solution for cryo-EM volume post-processing. *Commun. Biol.* **4**, 874 (2021).
18. R. Yan, *et al.*, Structural basis for sterol sensing by Scap and Insig. *Cell Rep.* **35**, 109299 (2021).
19. P. Emsley, B. Lohkamp, W. G. Scott, K. Cowtan, Features and development of Coot. *Acta Crystallogr D Biol Crystallogr* **66**, 486–501 (2010).
20. D. Liebschner, *et al.*, Macromolecular structure determination using X-rays, neutrons and electrons: recent developments in Phenix. *Acta Crystallogr D Struct Biol* **75**, 861–877 (2019).
21. T. I. Croll, ISOLDE: a physically realistic environment for model building into low-resolution electron-density maps. *Acta Crystallogr D Struct Biol* **74**, 519–530 (2018).
22. V. B. Chen, *et al.*, MolProbity: all-atom structure validation for macromolecular crystallography. *Acta Crystallogr D Biol Crystallogr* **66**, 12–21 (2010).
23. E. F. Pettersen, *et al.*, UCSF ChimeraX: Structure visualization for researchers, educators, and developers. *Protein Sci.* **30**, 70–82 (2021).

24. D. L. Kober, *et al.*, Identification of a degradation signal at the carboxy terminus of SREBP2: A new role for this domain in cholesterol homeostasis. *Proceedings of the National Academy of Sciences* **117**, 28080–28091 (2020).
25. M. R. McFarlane, *et al.*, Scap is required for sterol synthesis and crypt growth in intestinal mucosa. *J Lipid Res* **56**, 1560–1571 (2015).
26. J. L. Goldstein, S. K. Basu, M. S. Brown, Receptor-mediated endocytosis of low-density lipoprotein in cultured cells. *Meth Enzymol* **98**, 241–260 (1983).
27. M. S. Brown, J. R. Faust, J. L. Goldstein, I. Kaneko, A. Endo, Induction of 3-hydroxy-3-methylglutaryl coenzyme A reductase activity in human fibroblasts incubated with compactin (ML-236B), a competitive inhibitor of the reductase. *J Biol Chem* **253**, 1121–1128 (1978).
28. N. Sever, P. C. W. Lee, B.-L. Song, R. B. Rawson, R. A. DeBose-Boyd, Isolation of Mutant Cells Lacking Insig-1 through Selection with SR-12813, an Agent That Stimulates Degradation of 3-Hydroxy-3-methylglutaryl-Coenzyme A Reductase*. *J. Biol. Chem.* **279**, 43136–43147 (2004).
29. D. Yabe, Z.-P. Xia, C. M. Adams, R. B. Rawson, Three mutations in sterol-sensing domain of SCAP block interaction with insig and render SREBP cleavage insensitive to sterols. *Proc Natl Acad Sci USA* **99**, 16672–16677 (2002).
30. J. D. Feramisco, *et al.*, Intramembrane aspartic acid in SCAP protein governs cholesterol-induced conformational change. *Proc Natl Acad Sci USA* **102**, 3242–3247 (2005).
31. J. Jumper, *et al.*, Highly accurate protein structure prediction with AlphaFold. *Nature* **596**, 583–589 (2021).
32. D. N. Mastronarde, Automated electron microscope tomography using robust prediction of specimen movements. *J Struct Biol* **152**, 36–51 (2005).
33. S. K. Burley, *et al.*, Protein Crystallography, Methods and Protocols. *Methods Mol. Biol.* **1607**, 627–641 (2017).
